# Supplementary material for: Salmonella Typhimurium effector SseI regulates host peroxisomal dynamics to acquire lysosomal cholesterol
Source: EMBO Rep. 2024 Dec 18;26(3):656–89. doi: 10.1038/s44319-024-00328-x (PMC11811301; doi:10.1038/s44319-024-00328-x)
Supplement: Supplementary file 1 — Appendix [file 44319_2024_328_MOESM1_ESM.pdf]

APPENDIX

Table of content:

• Appendix Figure S1. Peroxisomes are required for efficient intracellular replication of STM……………………….Page 2

• Appendix Figure S2. Syt7 on SCV tethers PIP2 on peroxisome to facilitate cholesterol transfer…....Page 5

A.

Protein name	12 hour			6 hour			uninfected		
ACBD5	-0.36367	-2.03689	-0.00973	-0.0988	-0.19554	-0.03742	1.319934	1.387777	0.034344
PEX3	0.89978	0.469067	0.137543	0.114705	-0.99168	0.514217	-2.22436	0.822038	0.258692
PEX5	-0.79346	-1.33595	-0.87541	1.025249	1.429443	-0.79346	-0.1225	0.785969	0.680112
ABCD3	0.201949	0.209991	0.200756	1.818256	-1.02099	1.146192	-0.95603	-0.70415	-0.89597
PEX14	0.585097	1.009107	0.970482	-1.3168	0.864059	-1.67949	-0.61371	0.130819	0.050432
PEX11B	-0.67144	-1.15468	-1.07611	1.473764	0.215326	1.584201	-0.49893	0.25463	-0.12676
CAT	-0.00567	-0.02124	1.738799	-0.48532	-0.66178	1.441248	-0.31159	-0.25869	-1.43576
HACL1	-0.06049	0.078765	0.098409	-1.15766	-1.32611	1.133685	1.794817	0.049071	-0.61049
SCP2	-0.6713	-0.95753	-0.62629	0.115161	-0.39295	2.372697	0.445003	-0.46201	0.177219
GSTK1	0.85803	1.035536	0.895113	-1.67458	0.294689	-0.16209	-0.75179	-1.19225	0.697329
HSD17B4	1.220689	1.325433	1.259567	-0.54267	-0.95794	-0.91371	-0.30568	-1.0102	-0.07549
ACAA1	1.138537	0.861634	0.91076	-1.10227	-1.49901	-0.63767	0.096423	-0.66748	0.899079
FAR1	-0.79694	-0.81342	-1.07317	-0.86933	1.029626	-0.50121	0.777847	1.545097	0.701497
AGPS	1.089105	1.239158	1.158245	0.003042	0.152291	-0.69167	-1.30198	-1.26101	-0.38719
IDI1	0.777091	0.743887	0.620318	-2.34257	-0.65958	-0.21333	0.526102	0.096855	0.451232
PNPLA8	-0.66552	-0.43978	-0.68026	1.685896	0.251653	1.685896	-0.5629	-0.74578	-0.5292
USP9X	-0.37137	-0.7617	-0.54018	0.778428	0.185434	-1.93577	0.708586	0.632668	1.303909
IDE	0.122854	-0.0601	0.375697	0.073596	-1.52479	-0.5703	-0.45544	2.223272	-0.1848
IDH1	1.25686	1.228446	1.377689	-0.48653	-0.58868	-1.01593	-0.73216	-0.11904	-0.92065
ACAD11	-0.33696	-0.47953	-1.05496	1.206211	1.718446	0.353021	0.393415	-1.24392	-0.55572
ALDH3A2	1.193498	1.175148	1.138217	-0.18824	-0.58397	-0.77009	0.378024	-1.02501	-1.31758
PTGIS	-1.98282	-0.86835	-0.83793	0.29098	0.868225	0.894644	0.378235	0.770397	0.486625
SOD1	1.287824	1.299934	1.313835	-0.78367	-0.45272	-1.13939	-0.45237	-0.65386	-0.41959
TKT	1.271154	1.33617	1.353382	-0.78595	-0.52313	-0.82851	-0.37951	-0.68098	-0.76263
DNM1L	0.847368	0.758179	0.561466	-0.99465	-1.83204	-0.76918	0.733884	0.928211	-0.23324
BABAM2	-0.25169	-0.21993	-0.17204	1.572212	-0.39967	-1.03601	1.815503	-0.68424	-0.62414
PIK3R4	-0.37636	-0.25425	-0.33077	1.067532	-2.1393	1.055562	0.335465	-0.19622	0.838336
RAB8B	-1.12199	-1.57162	-1.18861	0.838114	0.713496	0.145089	0.87824	0.652212	0.655069
VIM	-1.2789	-1.37802	-1.23422	0.513847	1.206915	0.672226	0.387729	0.592196	0.518233
MED1	0.107333	0.143009	0.043653	0.014536	2.007155	0.435554	-1.73598	-0.17708	-0.83817
PPARA	-0.56978	-0.6477	-1.08908	-0.51896	-0.63262	1.212549	1.228092	1.484474	-0.46697

+2

-2

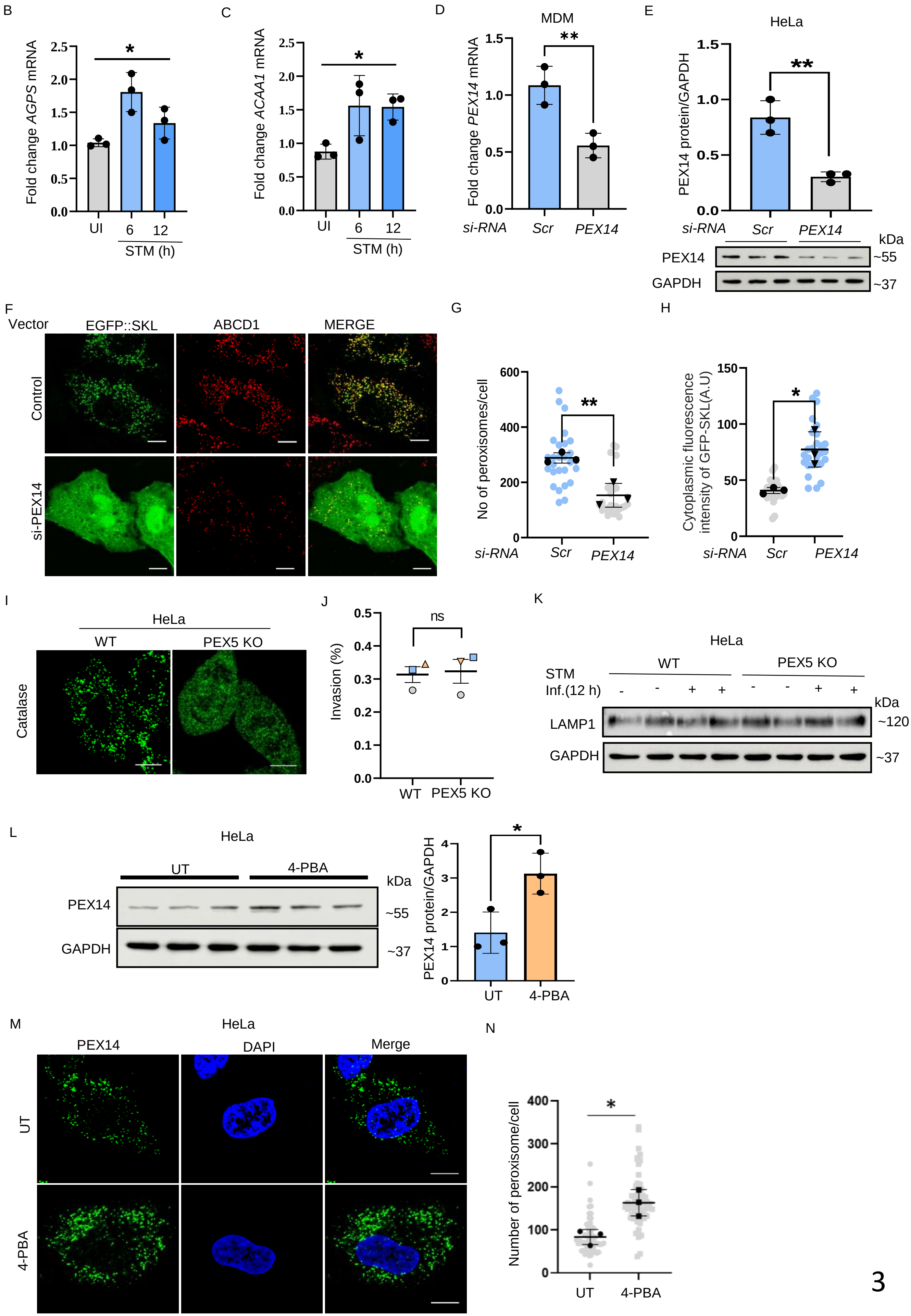
B
Fold change AGPS mRNA
2.5
2.0
1.5
1.0
0.5
0.0
*
UI
6
12
STM (h)
C
Fold change ACAA1 mRNA
*
STM (h)
D
MDM
Fold change PEX14 mRNA
**
si-RNA
Scr
PEX14
E
HeLa
PEX14 protein/GAPDH
**
kDa
PEX14
GAPDH
~55
~37
F
Vector
EGFP::SKL
ABCD1
MERGE
Control
si-PEX14
G
No of peroxisomes/cell
600
400
200
0
H
Cytoplasmic fluorescence intensity of GFP-SKL(A.U)
150
100
50
I
HeLa
WT
PEX5 KO
Catalase
J
Invasion (%)
ns
K
HeLa
STM
Inf.(12 h)
LAMP1
~120
L
HeLa
UT
4-PBA
PEX14 protein/GAPDH
M
HeLa
PEX14
DAPI
Merge
N
Number of peroxisome/cell
400
300

Appendix Figure S1: Peroxisomes are required for efficient intracellular replication of STM.

A. Z scale transformed values of peroxisome-related proteins identified by mass spectrometry after 6 and 12 h of STM infection in HeLa cells.

B-C. Fold change in mRNA levels of indicated peroxisomal genes (*AGPS* (A), *ACAA1 (B)* in HeLa cells post 6 and 12 h of STM infection compared with uninfected (UI). Statistics were performed on the mean of three independent experiments (black dots).

D. Graph representing the silencing efficiency of *PEX14* in MDM cells.

E. Immunoblot and densitometry analysis of pex14 protein levels after silencing PEX14 in HeLa cells. GAPDH is used as the loading control. The graph represents the ± SEM of three biological replicates.

F. Fluorescence microscopy images of EGFP::SKL in WT and *PEX14* KD HeLa cells stained with anti-ABCD1 (red) and EGFP (green) antibodies. Scale bar:10 µm.

G. Graph representing peroxisome number after silencing *PEX14* in HeLa cells.The graph represents the ± SEM of three independent experiments.

H. Graph representing cytoplasmic fluorescence intensity of EGFP::SKL.The graph represents the ± SEM of three independent experiments.

I. Fluorescence microscopy images of catalase in WT and *PEX5* KO HeLa cells stained with anti-Catalase (green) antibody. Scale bar:10 µm.

J. Graph representing percentage invasion of STM in *PEX5* knockout HeLa cells with WT HeLa cells. Statistics were performed on the mean of three independent experiments.

K. Immunoblot analysis of LAMP1 from whole cell lysates of WT and *PEX5* KO HeLa cells after STM infection for 12 h. GAPDH is used as the loading control.

L. Immunoblot and densitometry analysis of pex14 protein levels after treatment with 4-PBA (2-mM) in HeLa cells. GAPDH is used as the loading control. Graph represent the ± SEM of three biological replicates.

M. Fluorescence microscopy images and graph of peroxisomes in UT and after treatment with 4-PBA (2-mM) in HeLa cells for 10 days. HeLa cells stained with anti-PEX14 (green) and DAPI (Blue). Scale bar:10 µm.

N. Graph representing peroxisome number/cell after treatment with 4-PBA (2-mM) in HeLa cells. The graph represents the ± SEM of three independent experiments.

Data information: Data were analyzed using one-way ANOVA, Figures B (*p=0.0153) and C (*p=0.0450). Figures D-N data were analyzed using student's *t*-test Figures D (**p=0.0100), G (**p=0.0042), H (*p=0.0162), L (*p=0.0245), and N (*p=0.0174). 'ns' denotes no significant difference.

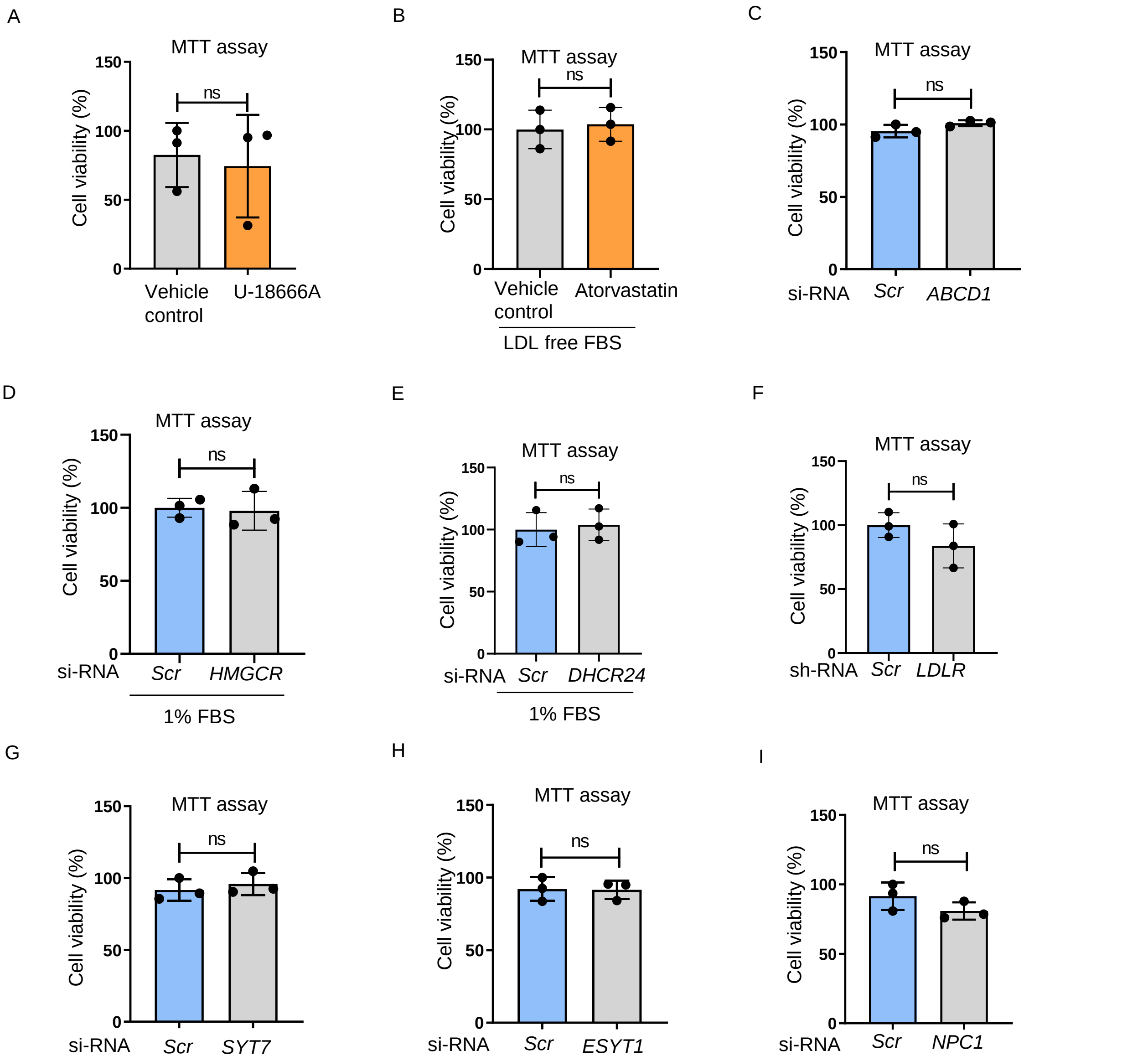

Appendix Figure S2: Syt7 on SCV tethers PIP2 on peroxisome to facilitate cholesterol transfer

A. Graph representing percentage cell viability of HeLa cells after treatment with U18666A measured by MTT assay. Graph represent the ± SEM of three biological replicates.

B. Graph representing percentage cell viability of HeLa cells after treatment with Atorvastatin measured by MTT assay. Graph represent the ± SEM of three biological replicates.

C. Graph representing percentage cell viability of HeLa cells after silencing *ABCD1* measured by MTT assay. Graph represent the ± SEM of three biological replicates.

D-I. Graph representing percentage cell viability of HeLa cells after silencing *HMGCR, DHCR24, LDLR, SYT7, ESYT1* and *NPC1* measured by MTT assay. Graph represent the ± SEM of three biological replicates.

Data information: In (A–I)-student's *t*-test, 'ns' denotes non-significant.
