# Supplementary figures and images for: Salmonella Typhimurium effector SseI regulates host peroxisomal dynamics to acquire lysosomal cholesterol
Source: EMBO Rep. 2024 Dec 18;26(3):656–89. doi: 10.1038/s44319-024-00328-x (PMC11811301; doi:10.1038/s44319-024-00328-x)

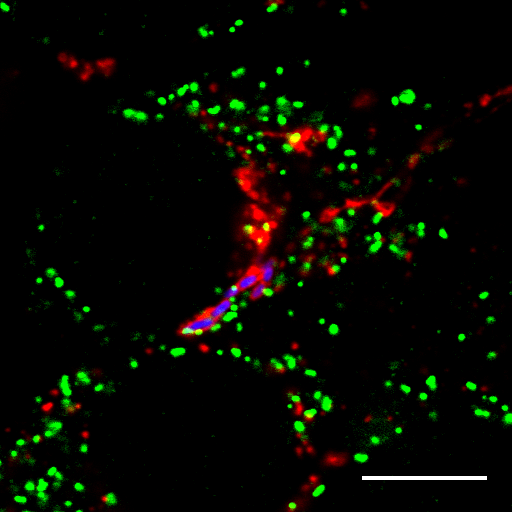

Supplement: Supplementary file 4 — Source data Fig. 1 [file 44319_2024_328_MOESM4_ESM.zip › Figure. 1/1A/A. 12H STM.tif]

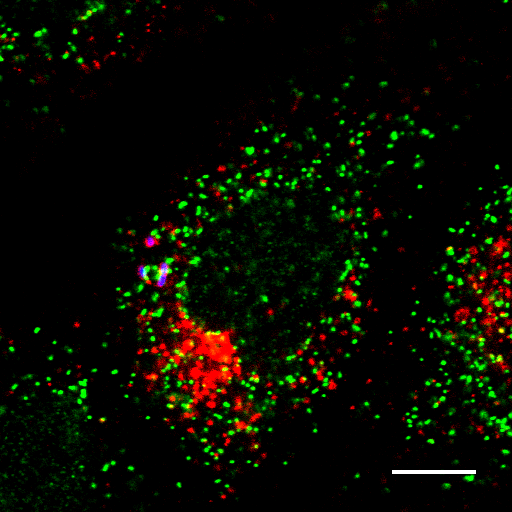

Supplement: Supplementary file 4 — Source data Fig. 1 [file 44319_2024_328_MOESM4_ESM.zip › Figure. 1/1A/A. 3H STM.tif]

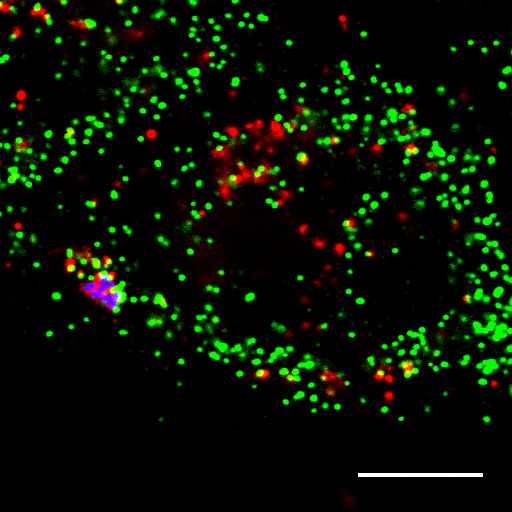

Supplement: Supplementary file 4 — Source data Fig. 1 [file 44319_2024_328_MOESM4_ESM.zip › Figure. 1/1A/A. 6H STM.tif]

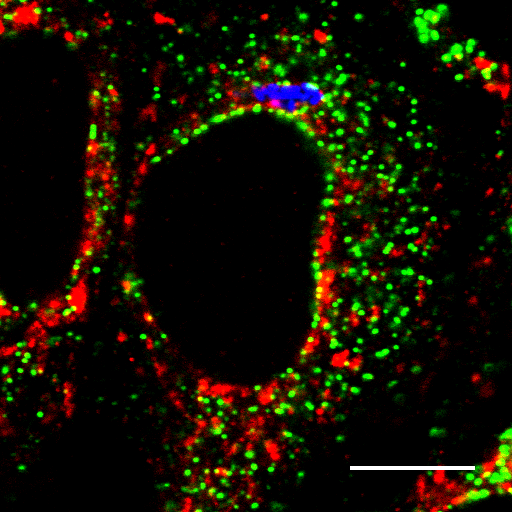

Supplement: Supplementary file 4 — Source data Fig. 1 [file 44319_2024_328_MOESM4_ESM.zip › Figure. 1/1B/B. 12H STM.tif]

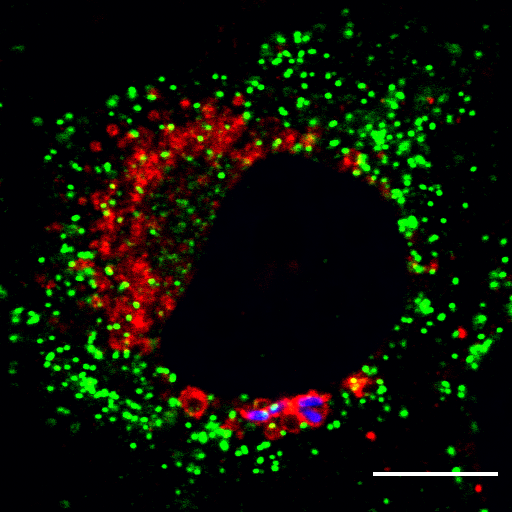

Supplement: Supplementary file 4 — Source data Fig. 1 [file 44319_2024_328_MOESM4_ESM.zip › Figure. 1/1B/B. 3H STM.tif]

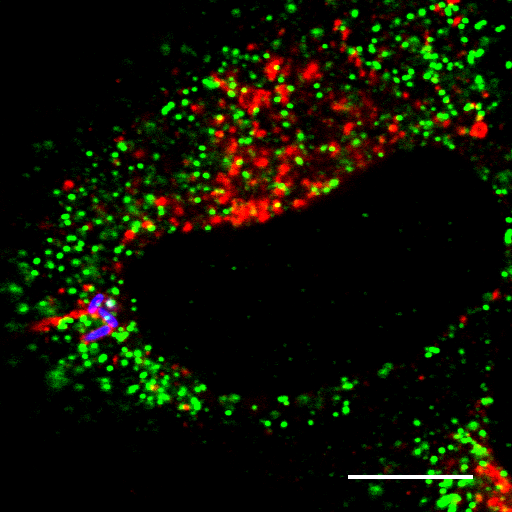

Supplement: Supplementary file 4 — Source data Fig. 1 [file 44319_2024_328_MOESM4_ESM.zip › Figure. 1/1B/B. 6H STM.tif]

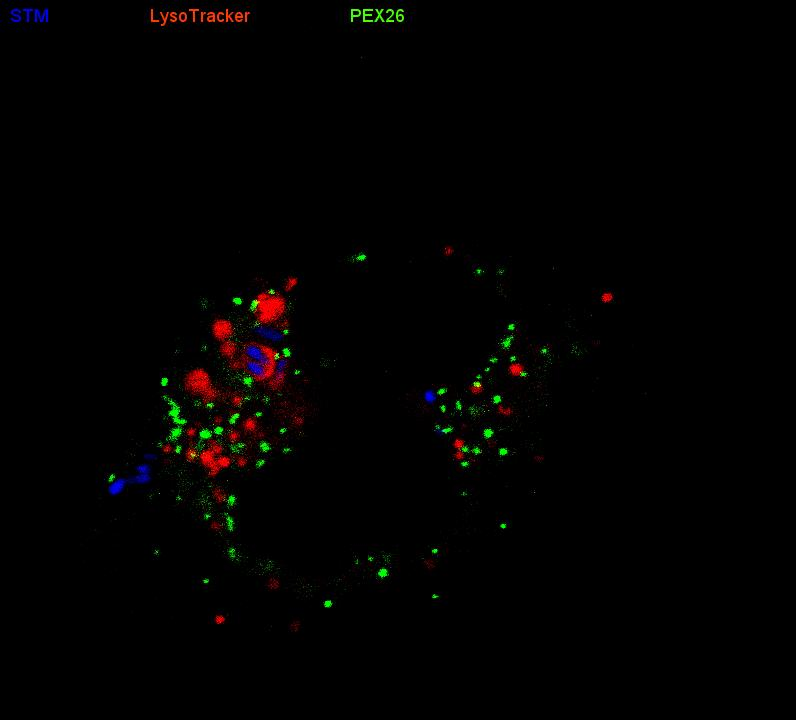

Supplement: Supplementary file 4 — Source data Fig. 1 [file 44319_2024_328_MOESM4_ESM.zip › Figure. 1/1E/E. live cell.tif]

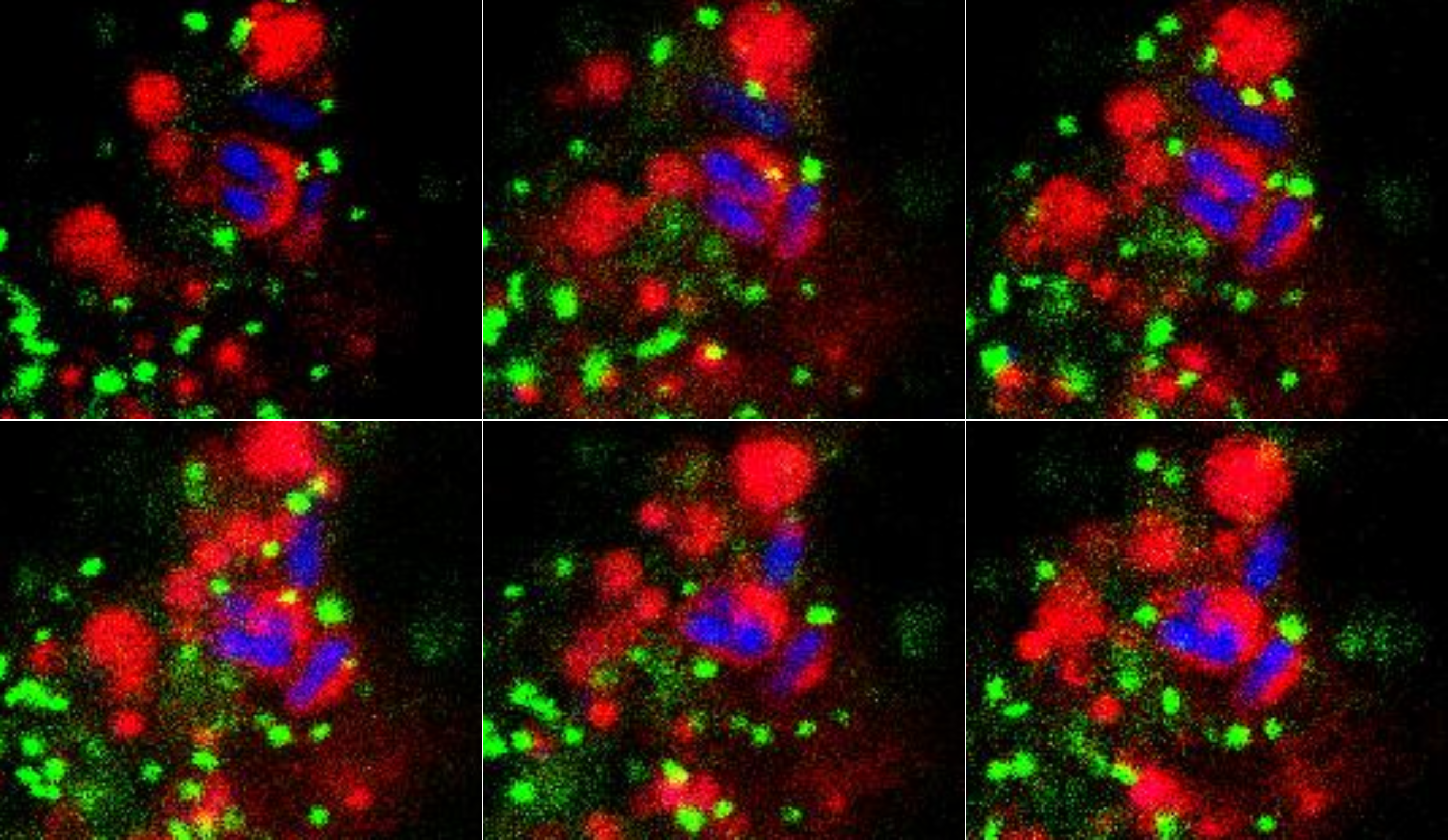

Supplement: Supplementary file 4 — Source data Fig. 1 [file 44319_2024_328_MOESM4_ESM.zip › Figure. 1/1E/Montage_zoom_lysoT.tif]

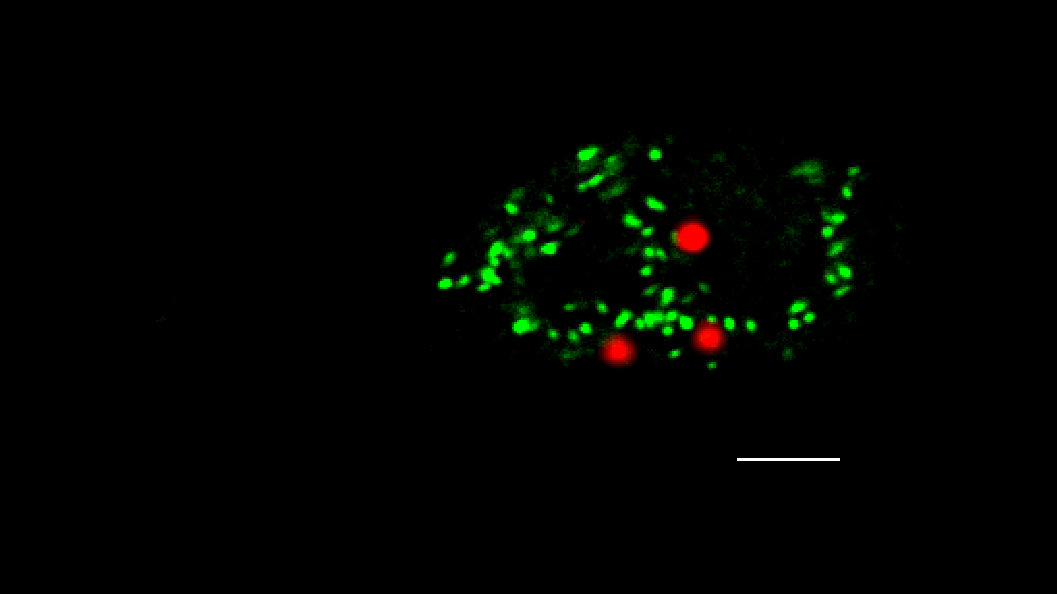

Supplement: Supplementary file 4 — Source data Fig. 1 [file 44319_2024_328_MOESM4_ESM.zip › Figure. 1/1F/Beads.tif]

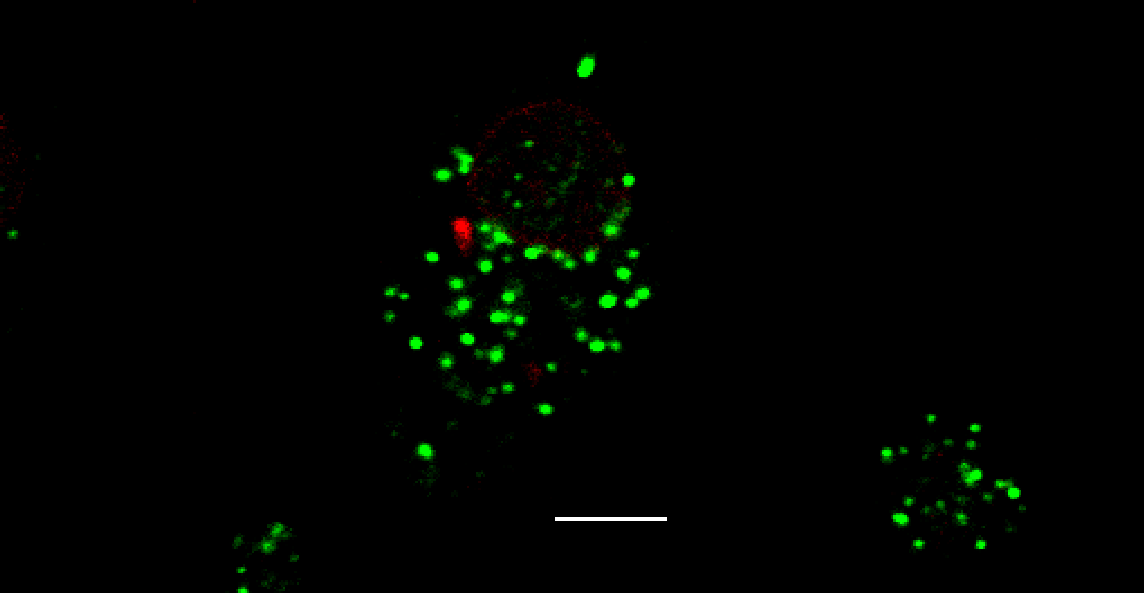

Supplement: Supplementary file 4 — Source data Fig. 1 [file 44319_2024_328_MOESM4_ESM.zip › Figure. 1/1F/Heat kill STM.tif]

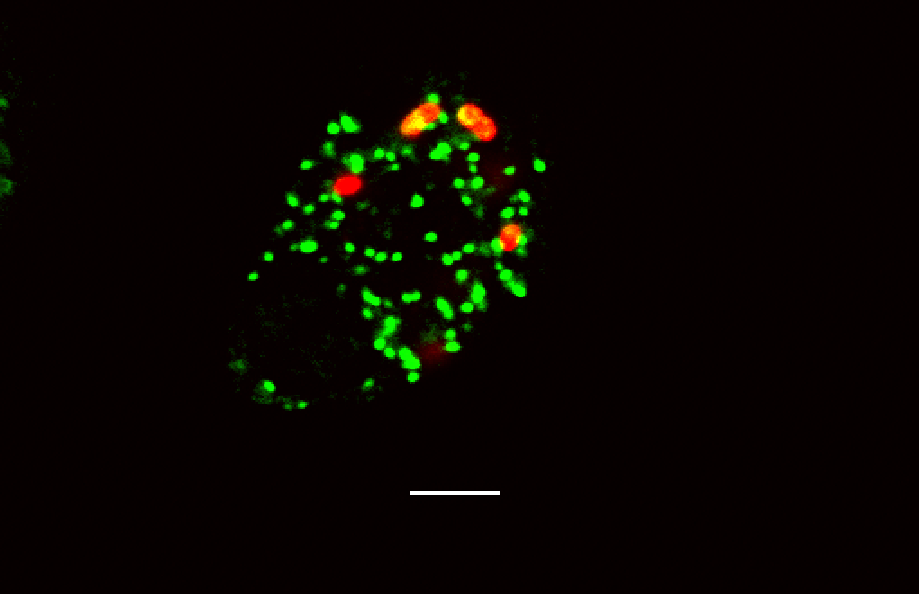

Supplement: Supplementary file 4 — Source data Fig. 1 [file 44319_2024_328_MOESM4_ESM.zip › Figure. 1/1F/LIVE STM.tif]

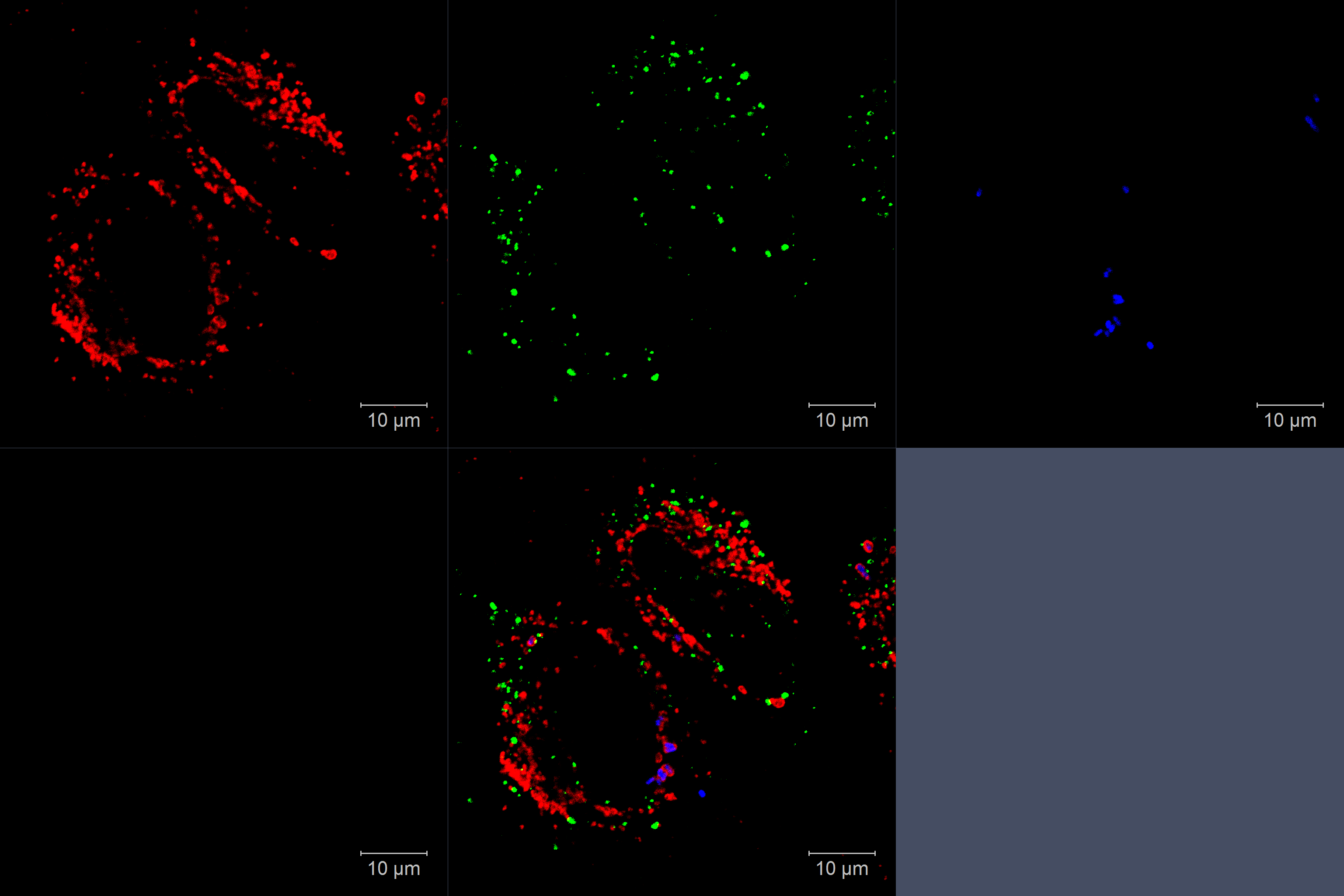

Supplement: Supplementary file 5 — Source data Fig. 2 [file 44319_2024_328_MOESM5_ESM.zip › Figure. 2/2K/PEX5 KO ..tif]

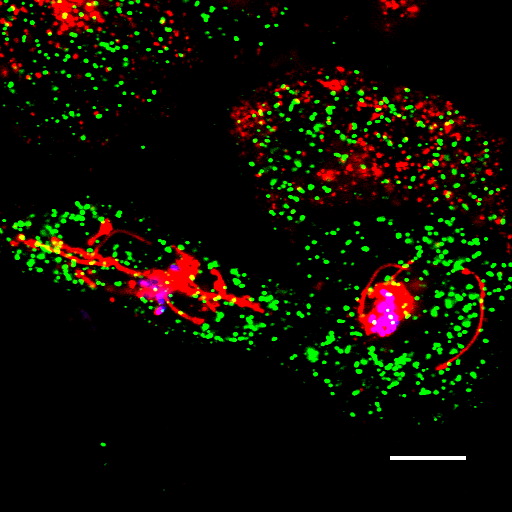

Supplement: Supplementary file 5 — Source data Fig. 2 [file 44319_2024_328_MOESM5_ESM.zip › Figure. 2/2K/WT..tif]

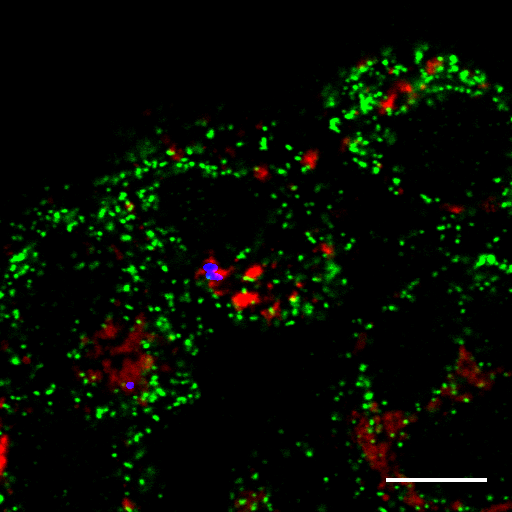

Supplement: Supplementary file 6 — Source data Fig. 3 [file 44319_2024_328_MOESM6_ESM.zip › Figure. 3/3A/A. SSEV..tif]

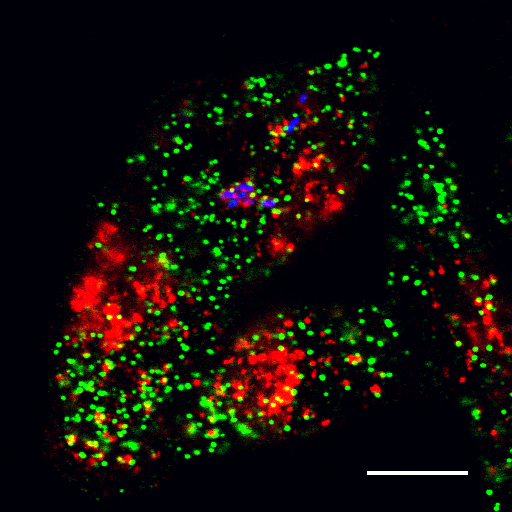

Supplement: Supplementary file 6 — Source data Fig. 3 [file 44319_2024_328_MOESM6_ESM.zip › Figure. 3/3A/A. WT..tif]

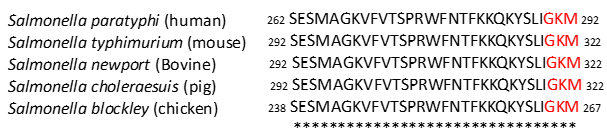

Supplement: Supplementary file 6 — Source data Fig. 3 [file 44319_2024_328_MOESM6_ESM.zip › Figure. 3/3B/GKM in SseI.tif]

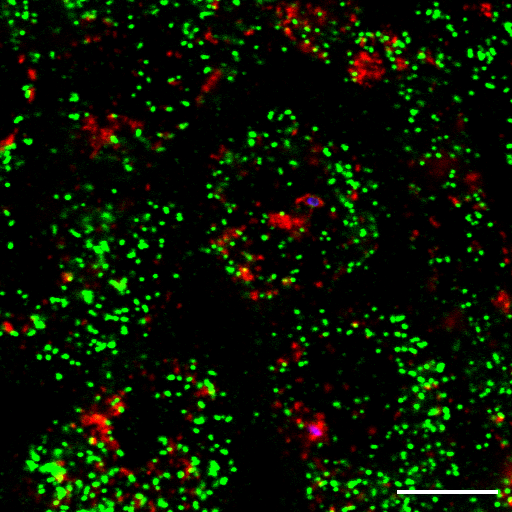

Supplement: Supplementary file 6 — Source data Fig. 3 [file 44319_2024_328_MOESM6_ESM.zip › Figure. 3/3C/C. SSEI.tif]

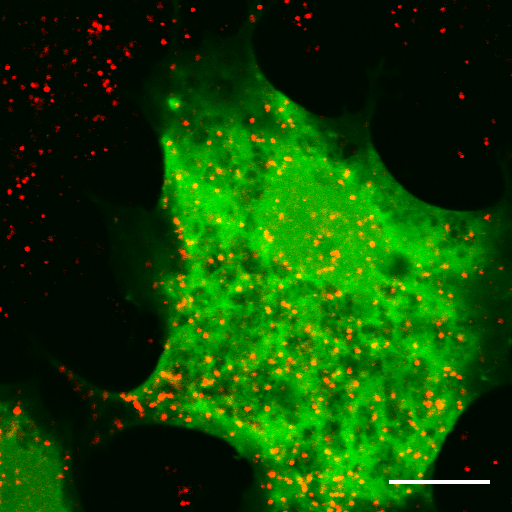

Supplement: Supplementary file 6 — Source data Fig. 3 [file 44319_2024_328_MOESM6_ESM.zip › Figure. 3/3E/E. EGFP-EV.tif]

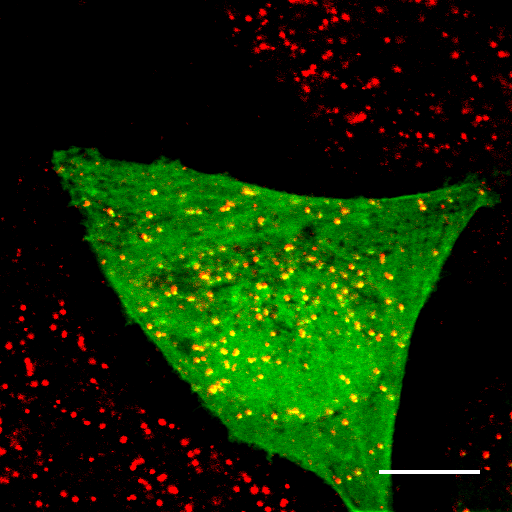

Supplement: Supplementary file 6 — Source data Fig. 3 [file 44319_2024_328_MOESM6_ESM.zip › Figure. 3/3E/E. EGFP-GKM.tif]

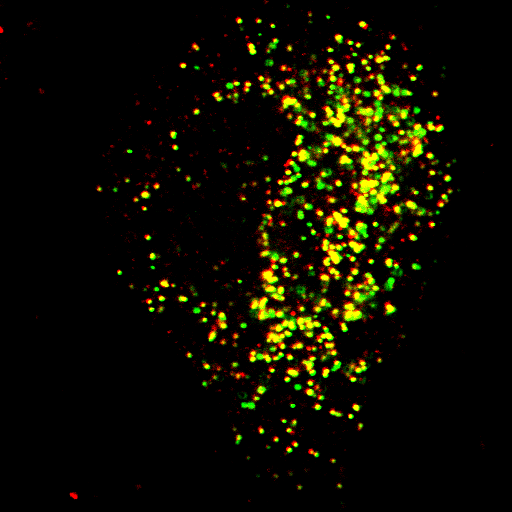

Supplement: Supplementary file 6 — Source data Fig. 3 [file 44319_2024_328_MOESM6_ESM.zip › Figure. 3/3E/E. EGFP-SKL..tif]

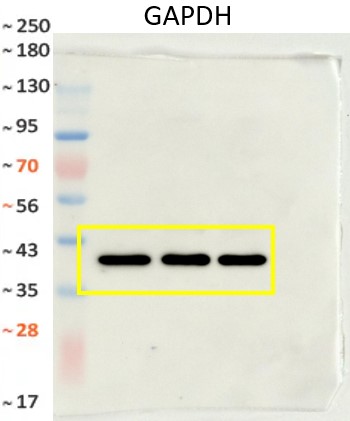

Supplement: Supplementary file 6 — Source data Fig. 3 [file 44319_2024_328_MOESM6_ESM.zip › Figure. 3/3H/H. GAPDH INPUT BLOT..jpg]

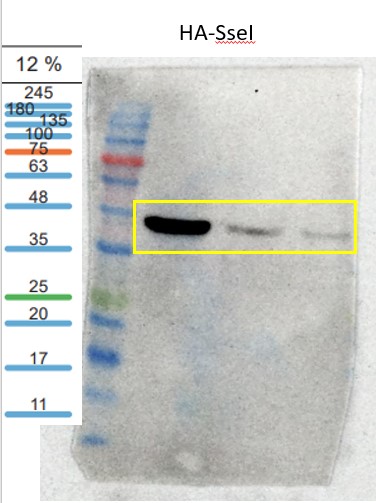

Supplement: Supplementary file 6 — Source data Fig. 3 [file 44319_2024_328_MOESM6_ESM.zip › Figure. 3/3H/H. HA-SseI BLOT.jpg]

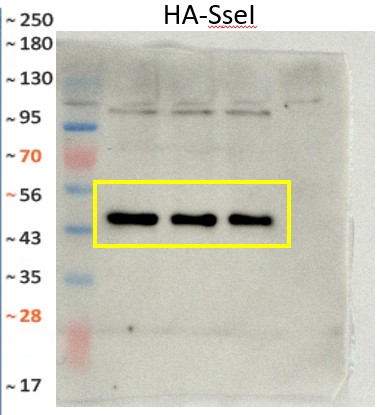

Supplement: Supplementary file 6 — Source data Fig. 3 [file 44319_2024_328_MOESM6_ESM.zip › Figure. 3/3H/H. HA-SseI INPUT BLOT.jpg]

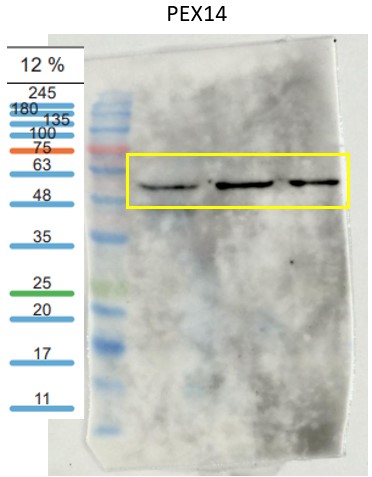

Supplement: Supplementary file 6 — Source data Fig. 3 [file 44319_2024_328_MOESM6_ESM.zip › Figure. 3/3H/H. PEX14 IP..jpg]

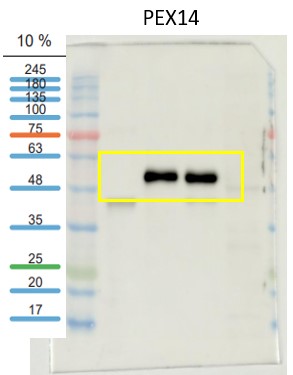

Supplement: Supplementary file 6 — Source data Fig. 3 [file 44319_2024_328_MOESM6_ESM.zip › Figure. 3/3J/J. PEX14 blot..jpg]

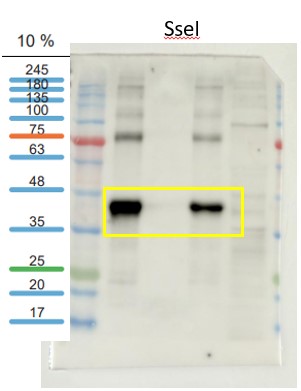

Supplement: Supplementary file 6 — Source data Fig. 3 [file 44319_2024_328_MOESM6_ESM.zip › Figure. 3/3J/J. SseI blot..jpg]

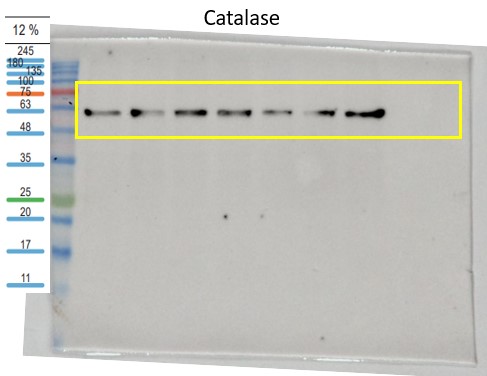

Supplement: Supplementary file 6 — Source data Fig. 3 [file 44319_2024_328_MOESM6_ESM.zip › Figure. 3/3K/K. Catalase .jpg]

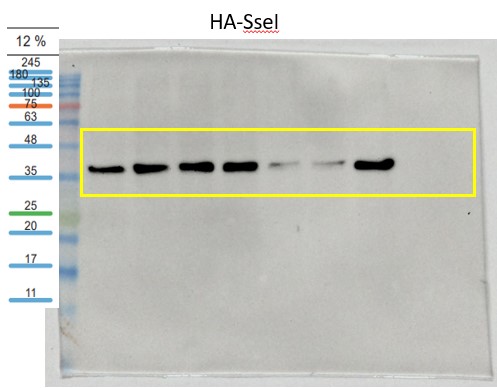

Supplement: Supplementary file 6 — Source data Fig. 3 [file 44319_2024_328_MOESM6_ESM.zip › Figure. 3/3K/K. HA-SseI.jpg]

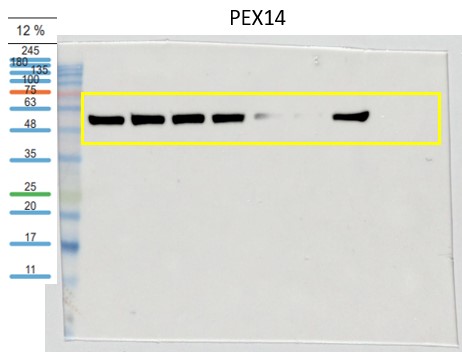

Supplement: Supplementary file 6 — Source data Fig. 3 [file 44319_2024_328_MOESM6_ESM.zip › Figure. 3/3K/K. PEX14.jpg]

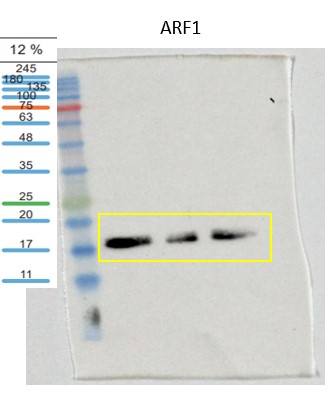

Supplement: Supplementary file 7 — Source data Fig. 4 [file 44319_2024_328_MOESM7_ESM.zip › Figure. 4/4A/A. ARF1 BLOT..jpg]

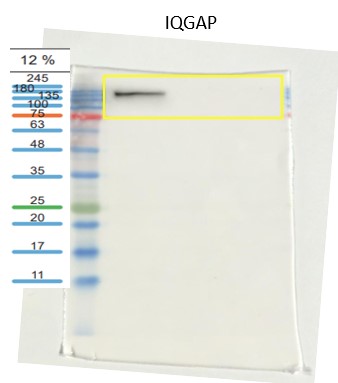

Supplement: Supplementary file 7 — Source data Fig. 4 [file 44319_2024_328_MOESM7_ESM.zip › Figure. 4/4A/A. IQGAP BLOT..jpg]

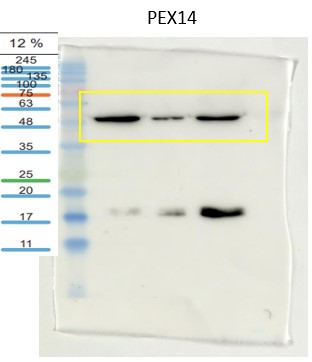

Supplement: Supplementary file 7 — Source data Fig. 4 [file 44319_2024_328_MOESM7_ESM.zip › Figure. 4/4A/A. PEX14 BLOT.jpg]

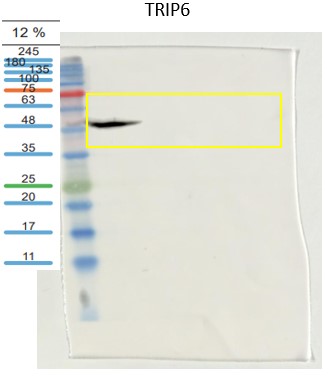

Supplement: Supplementary file 7 — Source data Fig. 4 [file 44319_2024_328_MOESM7_ESM.zip › Figure. 4/4A/A. TRIP6 BLOT.jpg]

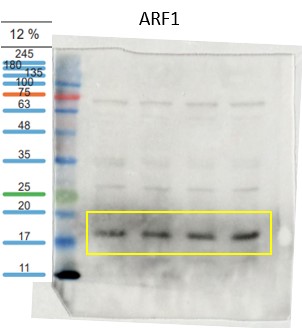

Supplement: Supplementary file 7 — Source data Fig. 4 [file 44319_2024_328_MOESM7_ESM.zip › Figure. 4/4B/B. ARF1 INPUT..jpg]

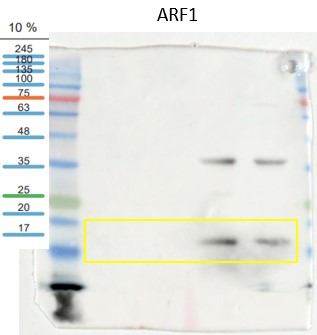

Supplement: Supplementary file 7 — Source data Fig. 4 [file 44319_2024_328_MOESM7_ESM.zip › Figure. 4/4B/B. ARF1 IP.jpg]

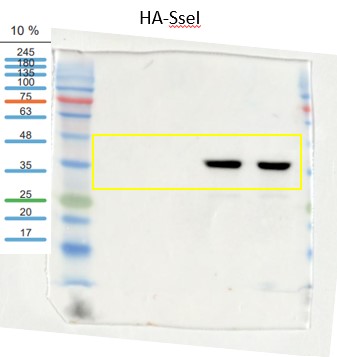

Supplement: Supplementary file 7 — Source data Fig. 4 [file 44319_2024_328_MOESM7_ESM.zip › Figure. 4/4B/B. HA-SseI BLOT.jpg]

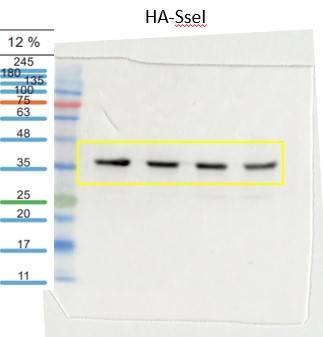

Supplement: Supplementary file 7 — Source data Fig. 4 [file 44319_2024_328_MOESM7_ESM.zip › Figure. 4/4B/B. HA-SseI input..jpg]

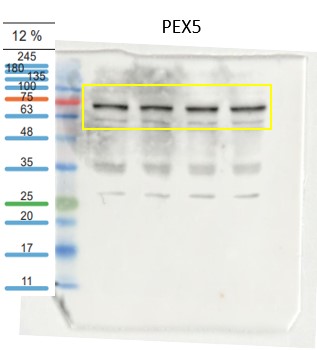

Supplement: Supplementary file 7 — Source data Fig. 4 [file 44319_2024_328_MOESM7_ESM.zip › Figure. 4/4B/B. PEX5 INPUT.jpg]

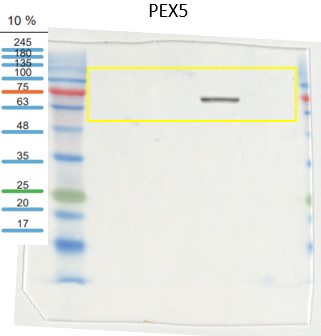

Supplement: Supplementary file 7 — Source data Fig. 4 [file 44319_2024_328_MOESM7_ESM.zip › Figure. 4/4B/B. PEX5 IP .jpg]

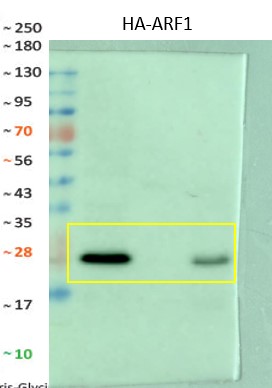

Supplement: Supplementary file 7 — Source data Fig. 4 [file 44319_2024_328_MOESM7_ESM.zip › Figure. 4/4C/C. HA-ARF1.jpg]

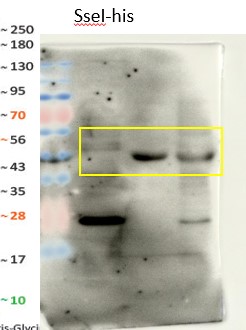

Supplement: Supplementary file 7 — Source data Fig. 4 [file 44319_2024_328_MOESM7_ESM.zip › Figure. 4/4C/C. SseI-HIS.jpg]

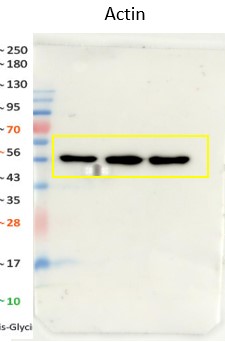

Supplement: Supplementary file 7 — Source data Fig. 4 [file 44319_2024_328_MOESM7_ESM.zip › Figure. 4/4D/D. Actin input blot..jpg]

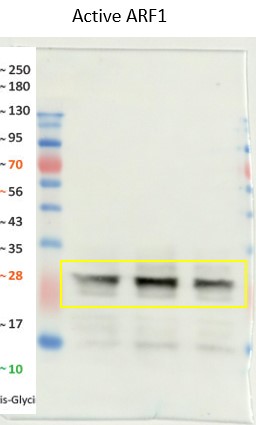

Supplement: Supplementary file 7 — Source data Fig. 4 [file 44319_2024_328_MOESM7_ESM.zip › Figure. 4/4D/D. Active ARF1.jpg]

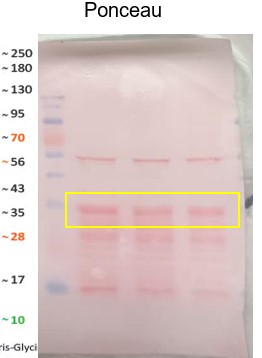

Supplement: Supplementary file 7 — Source data Fig. 4 [file 44319_2024_328_MOESM7_ESM.zip › Figure. 4/4D/D. Ponceau blot..jpg]

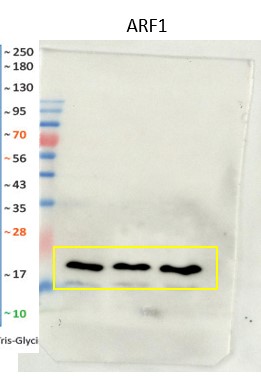

Supplement: Supplementary file 7 — Source data Fig. 4 [file 44319_2024_328_MOESM7_ESM.zip › Figure. 4/4D/D. Total ARF1 INPUT..jpg]

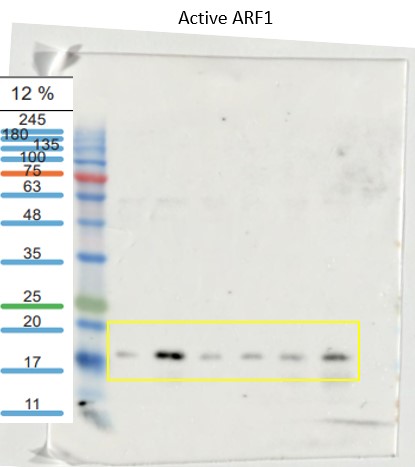

Supplement: Supplementary file 7 — Source data Fig. 4 [file 44319_2024_328_MOESM7_ESM.zip › Figure. 4/4F/F. Active ARF1.jpg]

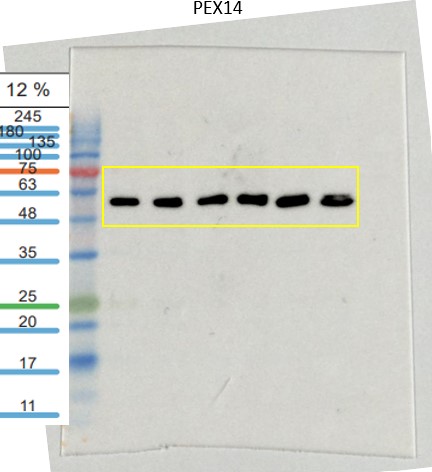

Supplement: Supplementary file 7 — Source data Fig. 4 [file 44319_2024_328_MOESM7_ESM.zip › Figure. 4/4F/F. PEX14 BLOT .jpg]

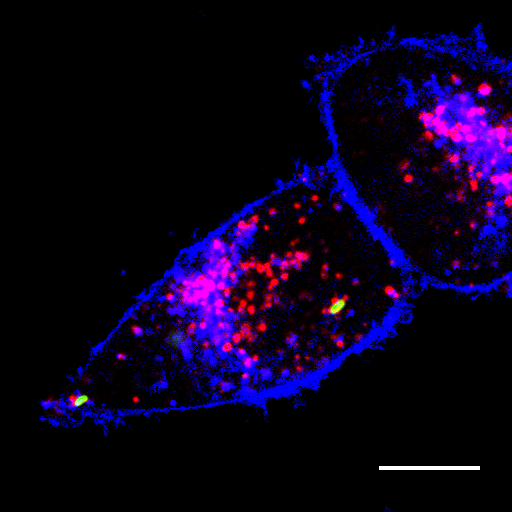

Supplement: Supplementary file 8 — Source data Fig. 5 [file 44319_2024_328_MOESM8_ESM.zip › Figure. 5/5C/C. PEX5 KO.tif]

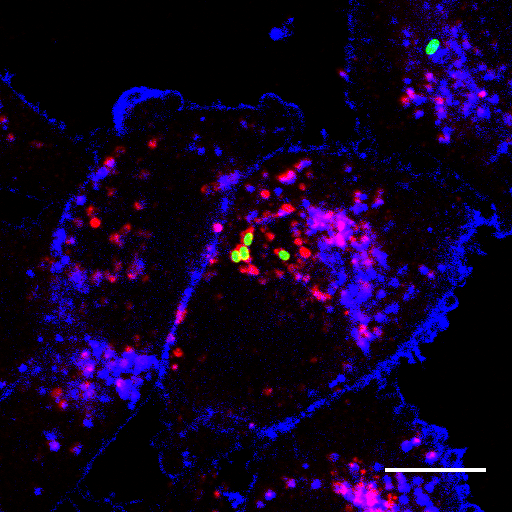

Supplement: Supplementary file 8 — Source data Fig. 5 [file 44319_2024_328_MOESM8_ESM.zip › Figure. 5/5C/C. SSEI.tif]

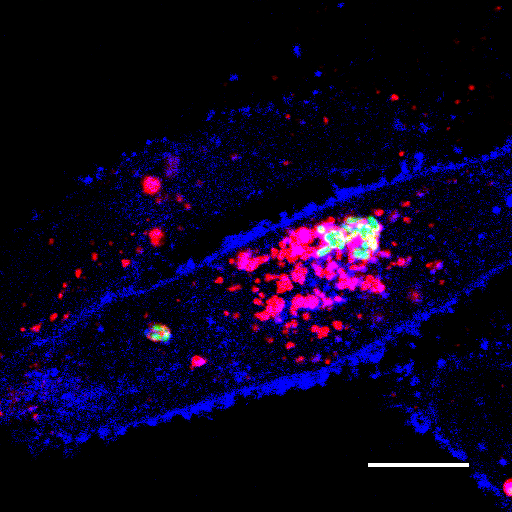

Supplement: Supplementary file 8 — Source data Fig. 5 [file 44319_2024_328_MOESM8_ESM.zip › Figure. 5/5C/C. WT.tif]

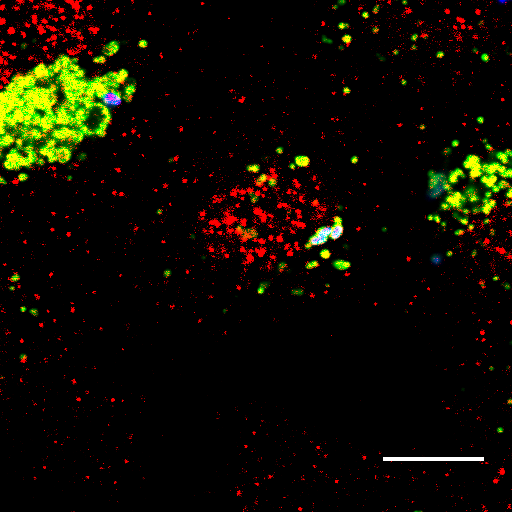

Supplement: Supplementary file 8 — Source data Fig. 5 [file 44319_2024_328_MOESM8_ESM.zip › Figure. 5/5I/WT STM + Syt-7 Flag.tif]

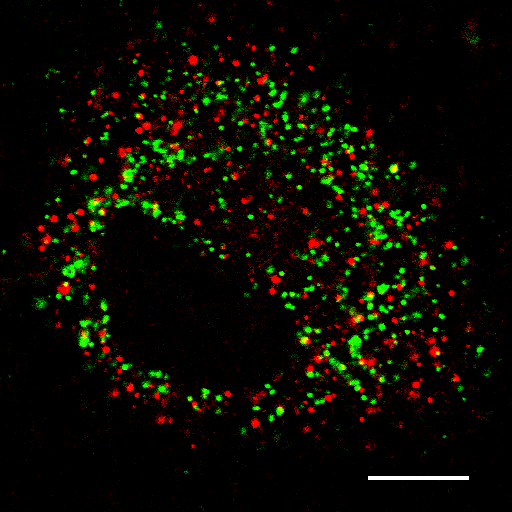

Supplement: Supplementary file 8 — Source data Fig. 5 [file 44319_2024_328_MOESM8_ESM.zip › Figure. 5/5J/HeLa cell With Syt-7 Uninfected.tif]

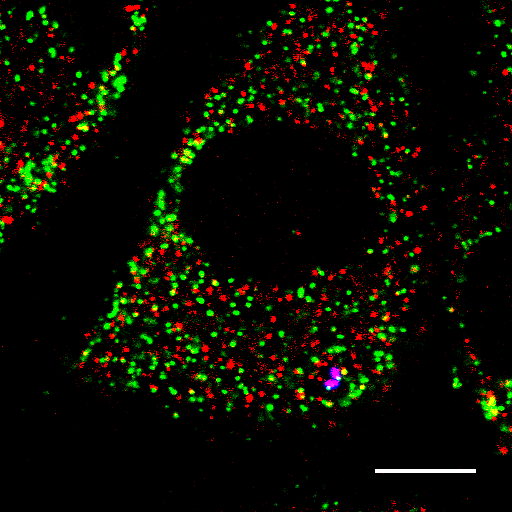

Supplement: Supplementary file 8 — Source data Fig. 5 [file 44319_2024_328_MOESM8_ESM.zip › Figure. 5/5K/WT STM + Syt-7 OE Flag.tif]

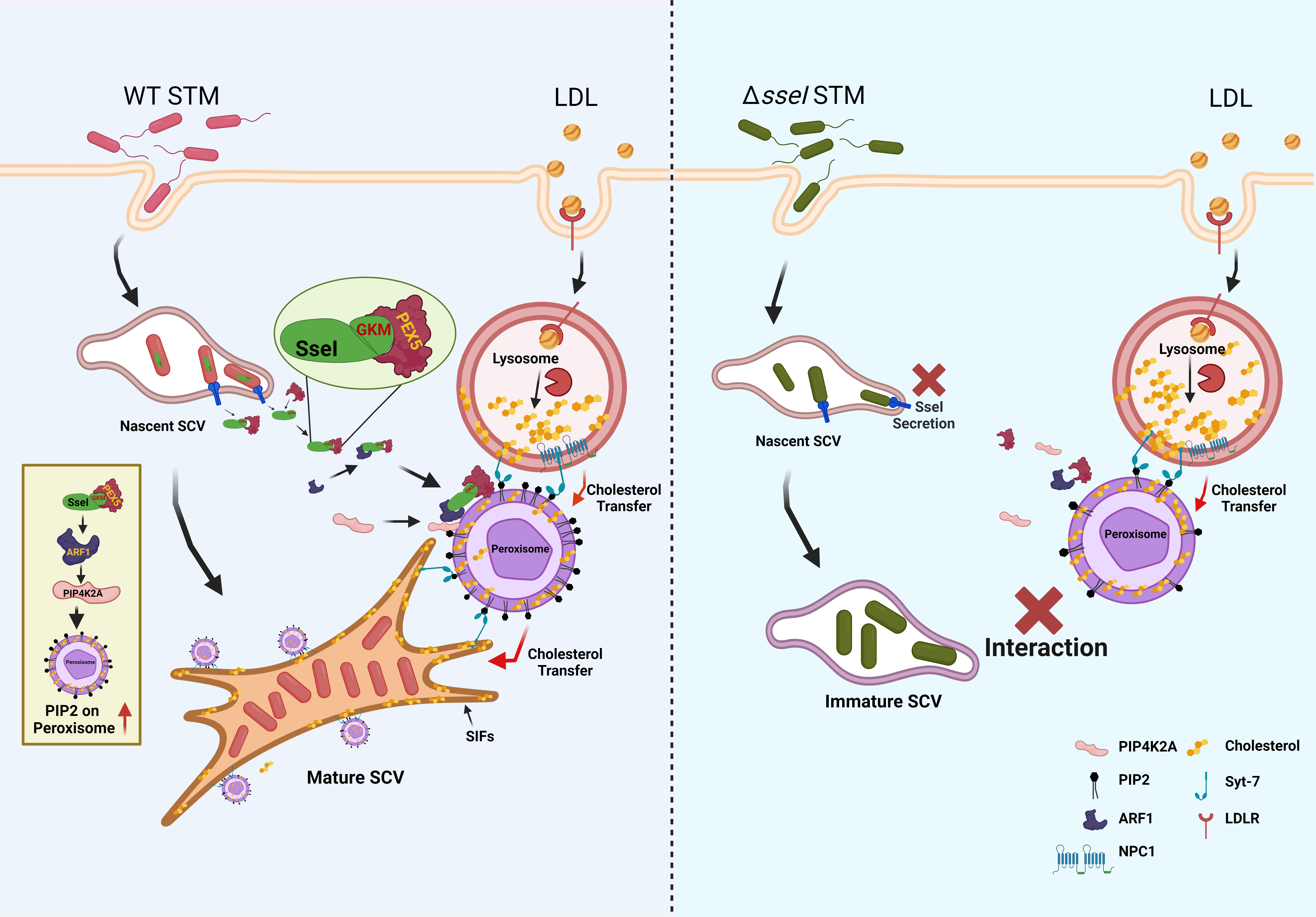

Supplement: Supplementary file 9 — Source data Fig. 6 [file 44319_2024_328_MOESM9_ESM.zip › Figure. 6/6G/Graphical Abstract.jpeg]
