# Supplementary material for: Salmonella Typhimurium effector SseI regulates host peroxisomal dynamics to acquire lysosomal cholesterol
Source: EMBO Rep. 2024 Dec 18;26(3):656–89. doi: 10.1038/s44319-024-00328-x (PMC11811301; doi:10.1038/s44319-024-00328-x)
Supplement: Supplementary file 10 — Expanded View Figures [file 44319_2024_328_MOESM10_ESM.pdf]

Expanded View Figures

Figure EV1. Peroxisomes are required for efficient intracellular replication of STM.

(**A**) Immunoblot and densitometry analysis of PEX14 protein levels in whole cell lysates of HeLa cells after STM infection for 3, 6, and 12 h. Actin served as a loading control. Densitometry analysis quantified PEX14 levels in STM-infected cells relative to uninfected controls with loading control actin. Graph represent the mean ± SEM of six independent experiments. (**B**) Immunoblot and densitometry analysis of PEX3 protein levels in whole cell lysates of HeLa cells after STM infection for 6 and 12 h. Actin served as a loading control. Densitometry analysis quantified PEX3 levels in STM-infected cells relative to uninfected controls. Graph represent the mean ± SEM of three independent experiments. (**C**, **D**) Fold change in mRNA levels of peroxisomal genes FAR1 (**C**) and HSD17B4 (**D**) in HeLa cells post 6 and 12 h of STM infection compared to uninfected controls. Graph represent the mean ± SEM of three independent experiments. (**E**) Immunoblot and densitometry analysis of *PEX14* protein levels after knockdown (KD) in HeLa cells. The membrane was probed with an anti-PEX14 antibody to compare protein levels in WT and PEX14 KD HeLa cells with loading control GAPDH. Graph represent the mean ± SEM of three biological replicates. (**F**) Immunoblot confirming the generation of PEX5 knockout (KO) HeLa cells by the CRISPR/Cas9 system. The membrane was probed with anti-PEX5 AND anti-GAPDH antibodies to compare protein levels in WT and PEX5 KO HeLa cells. (**G**) Fluorescence microscopy images of peroxisomes in WT and PEX5 KO HeLa cells stained with anti-LAMP1 (red) and anti-PEX14 (green) antibodies. Scale bar: 10 µm. (**H**) Fluorescence microscopy images of peroxisomes in WT and PEX5 KO HeLa transfected with EGFP::SKL construct and stained with anti-ABCD1 (red) antibodies. Scale bar: 10 µm. (**I**) Graph representing cytoplasmic fluorescence intensity of EGFP::SKL in WT HeLa cell and PEX5 KO Cells. Data represent the ± SEM of three independent experiments. (**J**) Fold change in PEX14 mRNA expression after treatment with 4-PBA (2 mM) in HeLa cells. Graph represent the ± SEM of four biological replicates. (**K**) Change in fluorescence intensity was used to monitor the replication of mCherry-labeled STM or *E. coli* HT115 in the intestine of control, and Prx-5 silenced *C. elegans* worms. Imaging of the nematode digestive tract was performed on Day 8 of adulthood. Fluorescence intensity measurements were obtained from approximately 30 worms per experimental group. (**L–N**) Graphs representing changes in cell viability after silencing *PEX14* (**L**), in PEX5 KO HeLa cells (**M**), and after treatment with 4-PBA (2 mM) (**N**) in HeLa cells, as determined by MTT assay. Graph represent the ± SEM of three biological replicates. Data information: Data were analyzed using one-way ANOVA, Figures (**A–C**) (**$p = 0.0015$), (**D**) (*$p = 0.0363$) and (**K**). 'ns' denotes no significant difference. Data were analyzed using the students' *t*-tests, Figures (**E**) (*$p = 0.0252$), (**I**) (****$p = 0.0001$), (**J**) (*$p = 0.0173$), (**L–N**), 'ns' denotes non-significant.

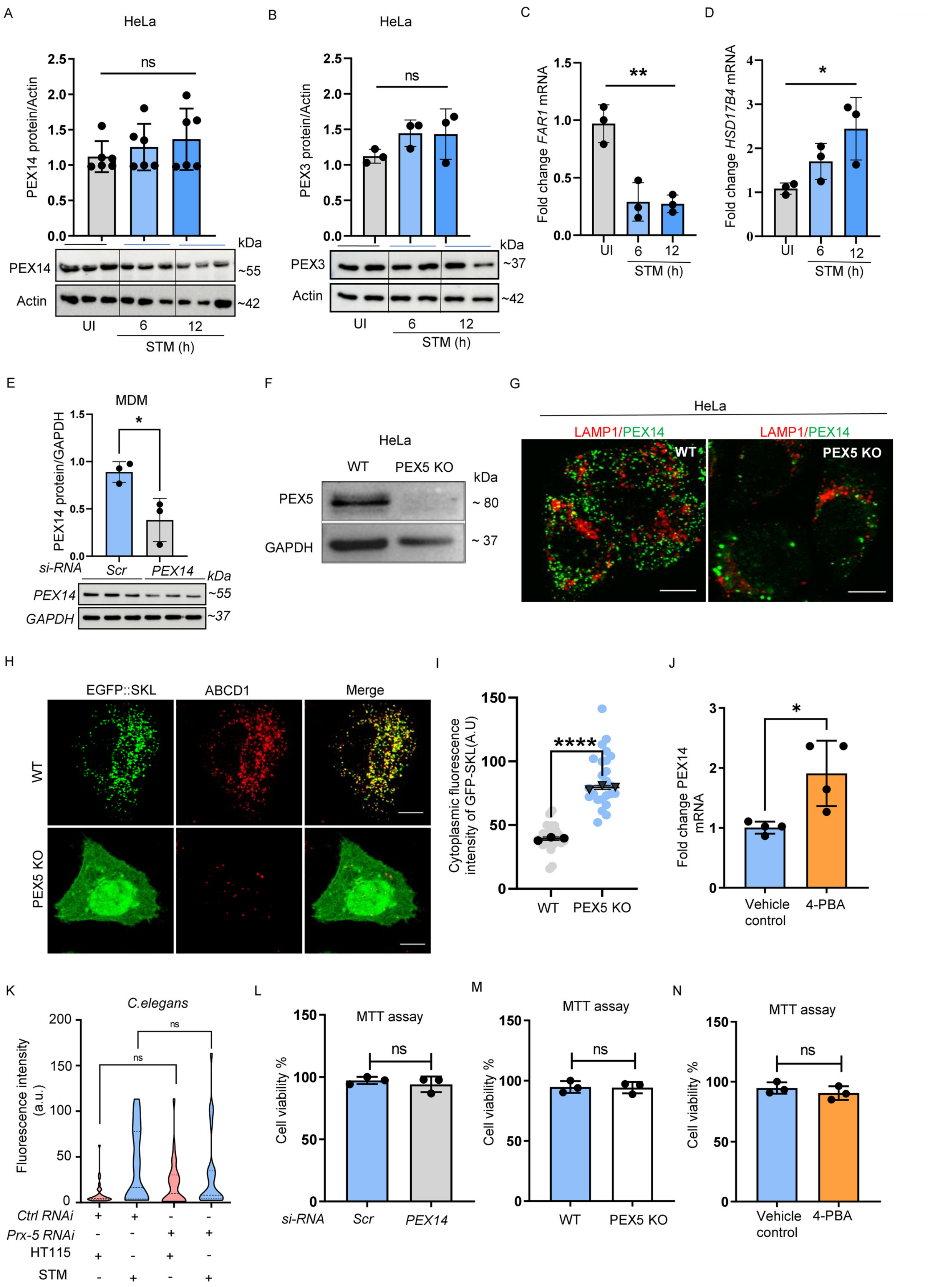

A
HeLa
PEX14 protein/Actin
ns
UI
6
12
STM (h)
PEX14
Actin
kDa
~55
~42
B
HeLa
PEX3 protein/Actin
ns
PEX3
Actin
~37
~42
C
Fold change FAR1 mRNA
**
D
Fold change HSD17B4 mRNA
*
E
MDM
PEX14 protein/GAPDH
si-RNA
Scr
PEX14
GAPDH
~55
~37
F
HeLa
WT
PEX5 KO
PEX5
GAPDH
~ 80
~ 37
G
HeLa
LAMP1/PEX14
WT
PEX5 KO
H
EGFP::SKL
ABCD1
Merge
WT
PEX5 KO
I
Cytoplasmic fluorescence intensity of GFP-SKL(A.U)
****
WT
PEX5 KO
J
Fold change PEX14 mRNA
*
Vehicle control
4-PBA
K
C.elegans
Fluorescence intensity (a.u.)
ns
Ctrl RNAi
Prx-5 RNAi
HT115
STM
L
MTT assay
Cell viability %
ns
si-RNA
Scr
PEX14
M
MTT assay
Cell viability %
ns
WT
PEX5 KO
N
MTT assay
Cell viability %
ns
Vehicle control
4-PBA

 © The Author(s)

A

S. No	Effectors	12 amino acid of C terminal	PTS1 software prediction
1	sseF	MDSSDHTSRGEP	Not targeted
2	sseG	LAQVTPEQRAPE	Not targeted
3	sseL	QTRRQIYEYSLQ	Not targeted
4	sseJ	LESFIAHHYSTE	Not targeted
5	SseI	FKKQKYSLIGKM	Twilight zone
6	pipB	QTLFNEFYSENI	Not targeted
7	steA	IKARYHNYLDNY	Not targeted
8	sifA	SEQQSGCLCCFL	Not targeted
9	pipB2	QTLFNEFYSENI	Not targeted
10	sifB	VETTKNEAHHQS	Not targeted
11	sopD2	ISEKSSCRNMLI	Not targeted
12	steB	HLLTCHSNGNVR	Not targeted
13	steC	LVAKVLKDELKK	Not targeted
14	slrP	KEVSSLMSAYWR	Not targeted
15	SspH2	LGSYLNVQWRRN	Not targeted
16	srfJ	GASTLLWRQESI	Not targeted
17	gtgA	TSPPRIAYEFSN	Not targeted
18	slrP	KEVSSLMSAYWR	Not targeted
19	sseA	APVEIPSGKTKR	Not targeted
20	SspB	PRGGRPALRVVK	Not targeted
21	SspA	TEAEREMRLGRG	Not targeted
22	SseD	TVLAEILRALTR	Not targeted
23	spvB	IKRIINMRVLNS	Not targeted
24	spvR	KLIQQELKQSTF	Not targeted
25	pipA	PSPPRMVYIFNK	Not targeted
26	gogA	TSPPRIAYESSN	Not targeted

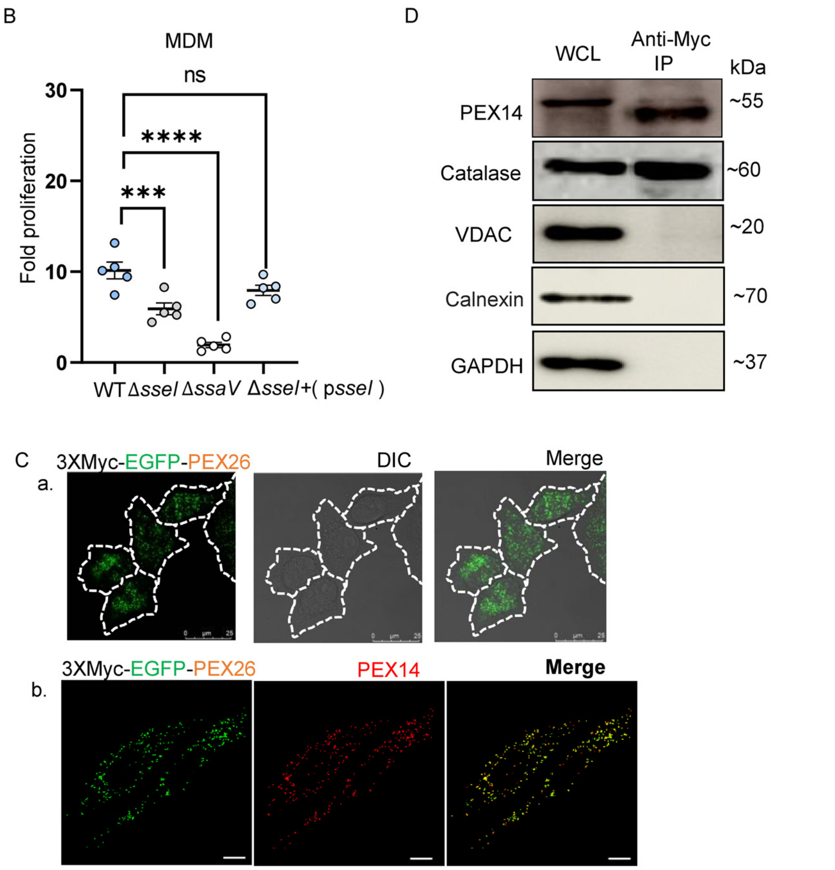

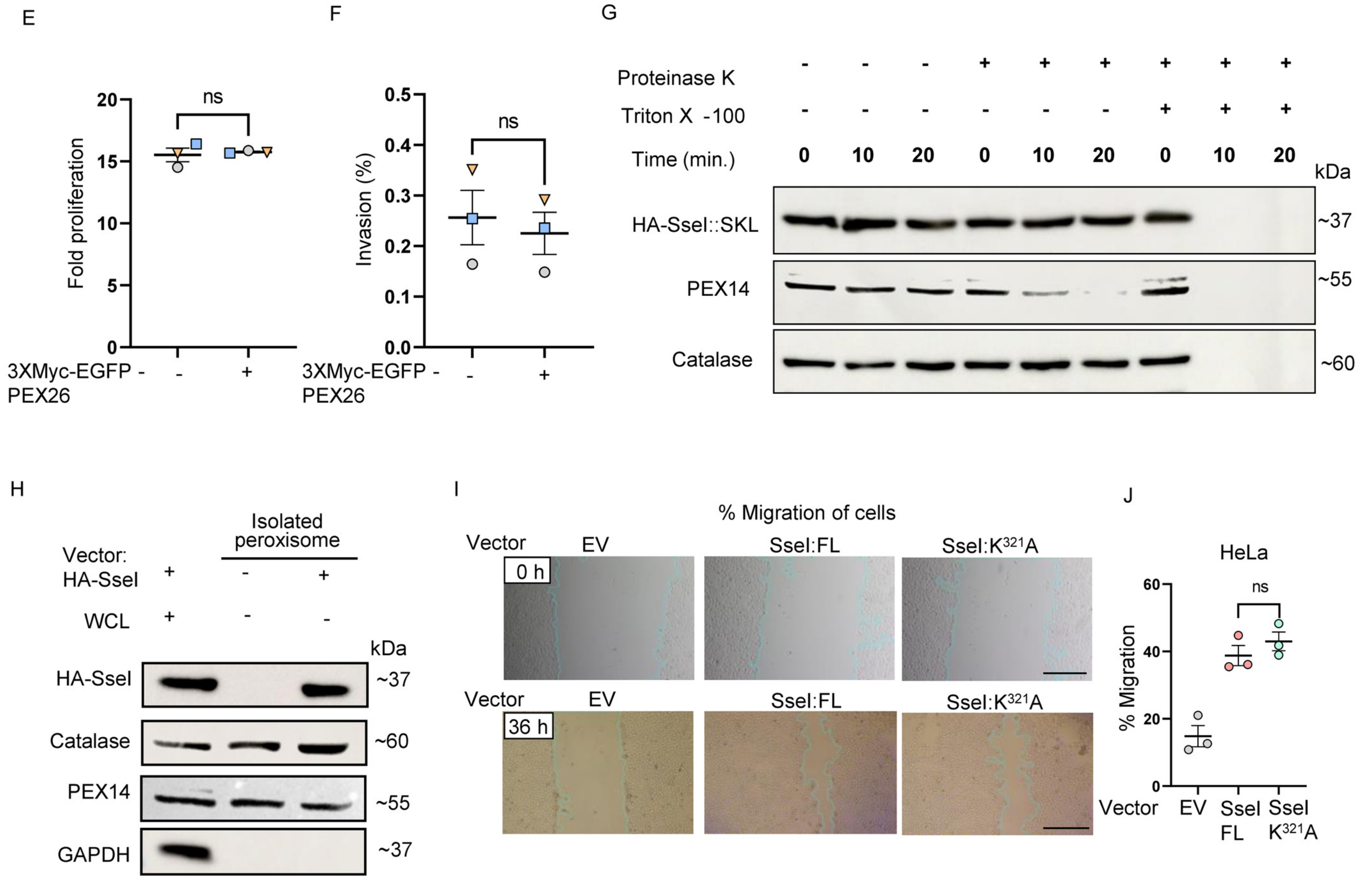

◀ **Figure EV2. Validation of PEROXO-tagged stable cell line expressing 3X myc-EGFP-PEX26.**

(**A**) Table listing key STM SPI2 effector proteins whose C-terminal amino acids (12 residues) were analyzed using in silico "PTS1 predictor" software to identify STM effector proteins containing the putative PTS1 motif. SseI is highlighted in green. (**B**) Fold proliferation of WT STM, Δ*ssaV* STM, Δ*sseI* STM, and Δ*sseI* STM complemented with pQE60-SseI in MDM cells. Each dot represents one donor; data are shown for five donors. (**C**) Fluorescence microscopy images validating the generation of a PEROXO-tagged stable cell line obtained by transfecting HeLa cells with lentivirus overexpressing 3xMyc-EGFP-PEX26 (a). Cells were stained with anti-PEX14 antibody to confirm the peroxisomal localization of the PEROXO tag green (3xMyc-EGFP-PEX26) (b). Scale bars: 25 μm (a), 10 μm (b). (**D**) Immunoblot analysis showing the purity of isolated peroxisomes using different organelle markers. PEX14, catalase, VDAC, calnexin, and GAPDH were used as markers for peroxisomes, mitochondria, ER, and cytosol, respectively. (**E**) Graph representing changes in fold proliferation of STM in PEROXO-tag (3xMyc-EGFP-PEX26) expressing stable HeLa cells. Data represent the mean ± SEM of three independent experiments. (**F**) Graph representing the percentage invasion of STM in PEROXO-tag (3xMyc-EGFP-PEX26) expressing stable HeLa cells. Data represent the mean ± SEM of three independent experiments. (**G**) Immunoblot analysis of HA-SseI::SKL protein after protease protection assay on isolated peroxisomes. Catalase and PEX14 were used as controls for matrix and membrane proteins, respectively. (**H**) Immunoblot analysis of HeLa cells were either transfected with SseI-HA or left untransfected. Whole cell lysate (WCL) and isolated peroxisome protein samples were subjected to immunoblot analysis using antibodies against HA, catalase, PEX14, and GAPDH. (**I**) Light microscopy images of HeLa cells during wound healing assay. HeLa cells were transfected with either full-length or $K^{321}A$ mutant SseI constructs. Scale bar: 10 μm. (**J**) Graph representing the percentage migration of HeLa cells after transfected with either full-length (FL) or $K^{321}A$ mutant of SseI constructs. Data information: Data were analyzed using one-way ANOVA, (**B**) (***$p = 0.0009$, ****$p = 0.0001$) and (**J**) 'ns' denotes no significant difference. (**E**, **F**) Data were analyzed using student's t-test, 'ns' denote non-significant.

 © The Author(s)

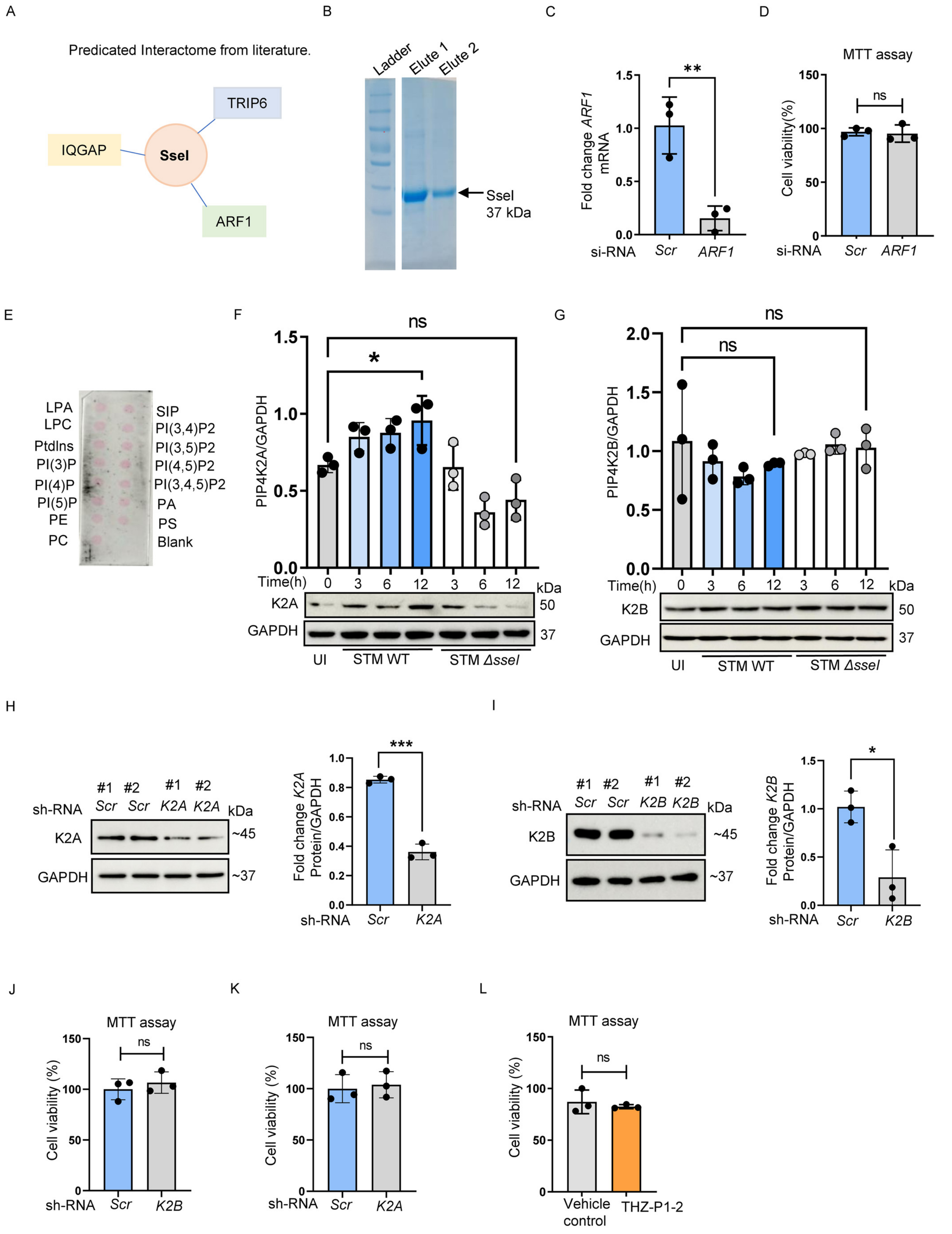
A
Predicated Interactome from literature.
TRIP6
IQGAP
SseI
ARF1
B
Ladder
Elute 1
Elute 2
SseI
37 kDa
C
Fold change ARF1 mRNA
si-RNA Scr ARF1
D
MTT assay
Cell viability(%)
si-RNA Scr ARF1
E
LPA
LPC
PtdIns
PI(3)P
PI(4)P
PI(5)P
PE
PC
SIP
PI(3,4)P2
PI(3,5)P2
PI(4,5)P2
PI(3,4,5)P2
PA
PS
Blank
F
PIP4K2A/GAPDH
Time(h)
K2A
GAPDH
UI
STM WT
STM ΔsseI
G
PIP4K2B/GAPDH
Time(h)
K2B
GAPDH
UI
STM WT
STM ΔsseI
H
sh-RNA
#1 #2 #1 #2
Scr Scr K2A K2A
K2A
GAPDH
Fold change K2A Protein/GAPDH
sh-RNA Scr K2A
I
sh-RNA
#1 #2 #1 #2
Scr Scr K2B K2B
K2B
GAPDH
Fold change K2B Protein/GAPDH
sh-RNA Scr K2B
J
MTT assay
Cell viability (%)
sh-RNA Scr K2B
K
MTT assay
Cell viability (%)
sh-RNA Scr K2A
L
MTT assay
Cell viability (%)
Vehicle control
THZ-P1-2

Figure EV3. SseI interacts with ARF1 to regulate its activity on the peroxisome membrane.

(**A**) Interactome of SseI with host proteins. Data retrieved from published literature. (**B**) Protein gel stained with Coomassie brilliant blue indicating purified His-tag SseI (~37 kDa) isolated from BL21 strain of *E. coli*. (**C**) Graph representing the silencing efficiency of *ARF1* in HeLa cells was validated using qPCR. Graph represent the ± SEM of three biological replicates. (**D**) Graph representing percentage cell viability of HeLa cells after silencing *ARF1* measured by MTT assay. Graph represent the ± SEM of four biological replicates. (**E**) PIP assay strip indicating protein-lipid overlay to identify the interaction of SseI with various indicated membrane lipids. Purified SseI protein was incubated on a lipid-coated membrane. A scheme of the PIP-strip membrane is presented. The red line highlights the phospholipid species tested. (**F**) Immunoblot and densitometry analysis of PIP4K2A protein levels after wild type (WT) or Δ*sseI* STM infection (MOI = 10) for 3, 6, and 12 h. 'UI' denotes uninfected cells. Densitometry analysis is from three biological replicates. (**G**) Immunoblot and densitometry analysis of PIP4K2B protein levels after wild type (WT) or Δ*sseI* STM infection (MOI = 10) for 3, 6, and 12 h. 'UI' denotes uninfected cells. Densitometry analysis is from three biological replicates. (**H**) Immunoblot analysis of PIP4K2A protein levels after silencing *PIP4K2A* in HeLa cells. GAPDH is used as the loading control. Graph represent the ±SEM of three biological replicates. (**I**) Immunoblot analysis of PIP4K2B protein levels after silencing *PIP4K2B* in HeLa cells. GAPDH is used as the loading control. Graph represent the ± SEM of three biological replicates. (**J**, **K**) Graph representing percentage cell viability of HeLa cells after silencing *PIP4K2A* (**J**) and *PIP4K2B* (**K**) in HeLa cells measured by MTT assay. Graph represent the ± SEM of three biological replicates. (**L**) Graph representing percentage cell viability of HeLa cells after treatment with THZ-P1-2 (1 μM) measured by MTT assay. Graph represent the ± SEM of three biological replicates. Data information: Data were analyzed using one-way ANOVA, (**F**) ($*p = 0.0274$), and (**G**) 'ns' denotes no significant difference. Data were analyzed using student's t-test, (**C**) ($**p = 0.0065$), (**D**, **H**) ($***p = 0.0001$), (**I**) ($*P = 0.0184$), (**J**–**L**), 'ns' denotes non-significant.

 © The Author(s)

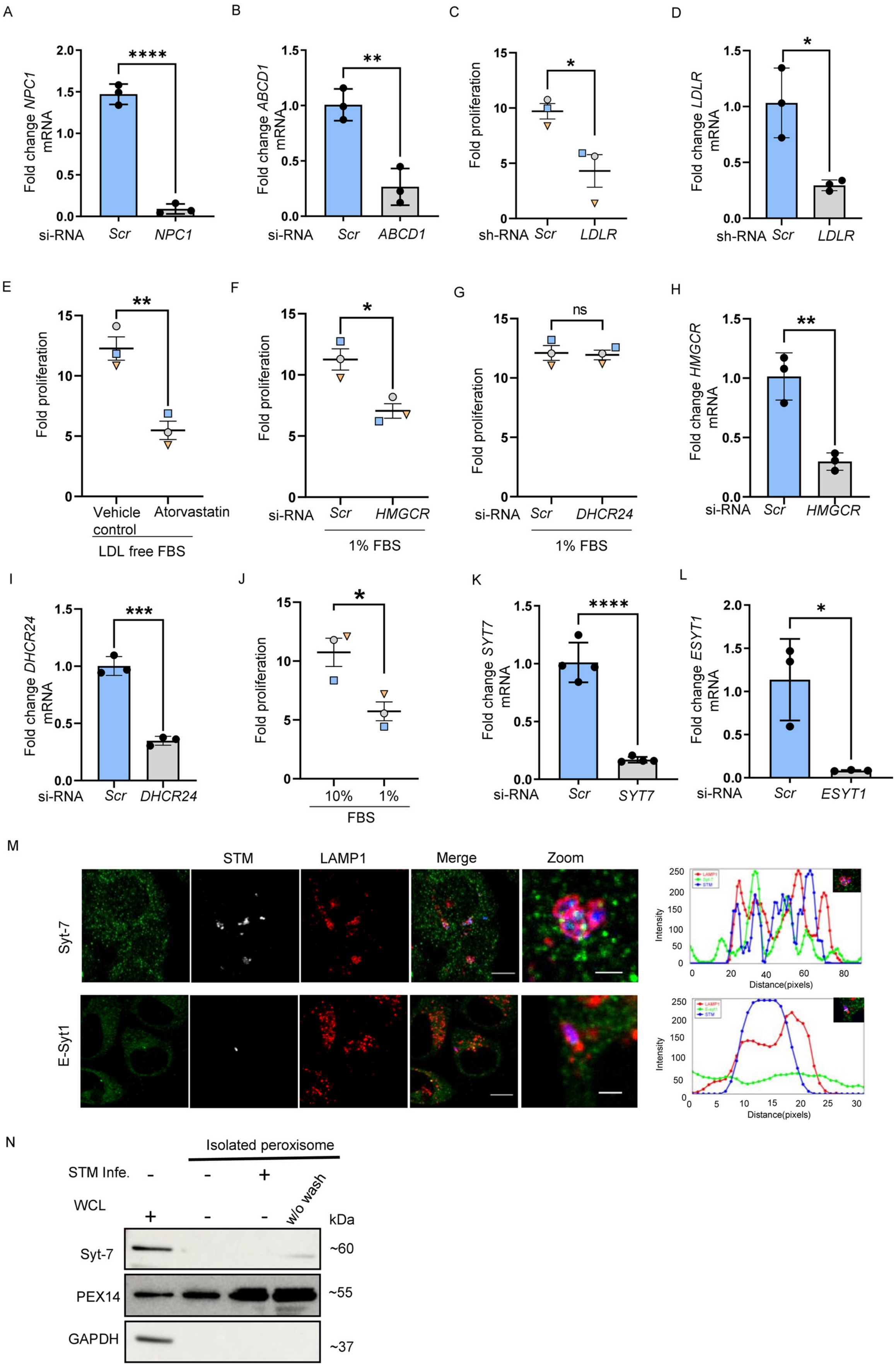
A
Fold change NPC1 mRNA
si-RNA Scr NPC1
****
B
Fold change ABCD1 mRNA
si-RNA Scr ABCD1
**
C
Fold proliferation
sh-RNA Scr LDLR
*
D
Fold change LDLR mRNA
sh-RNA Scr LDLR
*
E
Fold proliferation
Vehicle control Atorvastatin
LDL free FBS
**
F
Fold proliferation
si-RNA Scr HMGCR
1% FBS
*
G
Fold proliferation
si-RNA Scr DHCR24
1% FBS
ns
H
Fold change HMGCR mRNA
si-RNA Scr HMGCR
**
I
Fold change DHCR24 mRNA
si-RNA Scr DHCR24
***
J
Fold proliferation
10% 1%
FBS
*
K
Fold change SYT7 mRNA
si-RNA Scr SYT7
****
L
Fold change ESYT1 mRNA
si-RNA Scr ESYT1
*
M
STM
LAMP1
Merge
Zoom
Syt-7
E-Syt1
Intensity
Distance(pixels)
N
Isolated peroxisome
STM Infe.
WCL
w/o wash
kDa
Syt-7
PEX14
GAPDH
~60
~55
~37

Figure EV4. Syt7 on SCV tethers PIP2 on peroxisome to facilitate cholesterol transfer.

(**A**) Fold change in mRNA levels of *NPC1* after its silencing in HeLa cells. Graph represent the ± SEM of three biological replicates. (**B**) Fold change in mRNA levels of *ABCD1* after its silencing in HeLa cells. Graph represent the ± SEM of three biological replicates. (**C**) Graph representing the changes in fold proliferation of STM (MOI = 10) after silencing *LDLR* in HeLa cells. Three independent experiments were performed. Each dot represents the mean of an independent experiment. (**D**) Fold change in mRNA levels of *LDLR* after its silencing in HeLa cells. Graph represent the ± SEM of three biological replicates. (**E**) Fold proliferation of STM (MOI = 10) in HeLa cells under LDL-free FBS conditions following Atorvastatin (1 µM). HeLa cells were treated with Atorvastatin and DMSO as Vehicle control. Three independent experiments were performed. Each dot represents the mean of an independent experiment. (**F**) Fold proliferation of STM (MOI = 10) in HeLa cells under 1% FBS conditions following HMGCR silencing. HeLa cells were transfected with siRNA targeting *HMGCR* or scrambled siRNA (Scr) control. Three independent experiments were performed. Each dot represents the mean of an independent experiment. (**G**) Graph representing the changes in fold proliferation of STM (MOI = 10) after silencing *DHCR24* in HeLa cells at 1% FBS condition. Three independent experiments were performed. Each dot represents the mean of an independent experiment. (**H**) Fold change in mRNA levels of *HMGCR* after its silencing in HeLa cells. Graph represent the ± SEM of three biological replicates. (**I**) Fold change in mRNA levels of *DHCR24* after its silencing in HeLa cells. Graph represent the ± SEM of three biological replicates. (**J**) Graph representing the changes in intracellular fold proliferation of STM (MOI = 10) in HeLa cells at 10% and 1% FBS condition. Three independent experiments were performed. Each dot represents the mean of an independent experiment. (**K**) Fold change in mRNA levels of *SYT7* after its silencing in HeLa cells. Graph represent the ± SEM of three biological replicates. (**L**) Fold change in mRNA levels of *ESYT1* after its silencing in HeLa cells. Graph represent the ± SEM of three biological replicates. (**M**) Representative microscopy images of HeLa cells infected with GFP-tagged STM (blue) (MOI = 50) and immunostained for endogenous Syt-7/E-syt1 (green) and LAMP1 (red) antibody. The intensity profile indicating the overlap of signals of Syt-7-LAMP1 and E-syt1-LAMP1 is shown on the right. Scale bars: 10 µm, 2 µm. (**N**) Immunoblot Analysis of Syt-7 Localization on Peroxisomes in HeLa cells. Cells were either infected with STM or left uninfected. Whole-cell lysates (WCL) and isolated peroxisome protein samples (both washed and unwashed) were subjected to immunoblot analysis using antibodies against Syt-7, the peroxisomal marker PEX14, and GAPDH as a loading control. Data information: (**A–F**) Data were analyzed using student's t-test; (**A**) (****$p = 0.0001$), (**B**) (**$p = 0.0043$), (**C**) (*$p = 0.0297$), (**D**) (*$p = 0.0156$), (**E**) (**$p = 0.0053$), (**F**) (*$p = 0.0160$), (**G**, **H**) (**$p = 0.0043$), (**I**) (***$p = 0.0002$), (**J**) (*$p = 0.0253$), and (**K**) (****$p = 0.0001$), (**L**) (*$p = 0.0182$), 'ns' denotes non-significant.

© The Author(s)
